# Supplementary material for: The photometric variability of massive stars due to gravity waves excited by core convection
Source: Nat Astron. 2023 Jul 27;7(10):1228–34. doi: 10.1038/s41550-023-02040-7 (PMC10581898; doi:10.1038/s41550-023-02040-7)
Supplement: Supplementary file 1 — Supplementary Figs. 1–13, Tables 1–3, Derivations, Implementation Details and Discussion. [file 41550_2023_2040_MOESM1_ESM.pdf]

nature astronomy

Article https://doi.org/10.1038/s41550-023-02040-7

The photometric variability of massive stars due to gravity waves excited by core convection

In the format provided by the authors and unedited

Draft version June 22, 2023
Typeset using LaTeX default style in AASTeX631

Supplemental Materials for "The photometric variability of massive stars due to gravity waves excited by core convection"

Evan H. Anders, Daniel Lecoanet, Matteo Cantiello, Keaton J. Burns, Benjamin A. Hyatt, Emma Kaufman, Richard H. D. Townsend, Benjamin P. Brown, Geoffrey M. Vasil, Jeffrey S. Oishi, and Adam S. Jermyn

Contents

1. MESA Stellar Models 2
1.1. Near-ZAMS calculations used in this work 2
1.2. Input physics 2

2. Dedalus Equations & Simulation Details 2
2.1. Equation formulation 2
2.2. Boundary conditions 3
2.3. Nondimensionalization 4
2.4. Discretization and timestepping choices 4
2.5. Damping layers 5
2.6. Constructing the background state from MESA models 5
2.7. Table of simulations 7

3. The Wave Luminosity Spectrum in Wave Generation Simulations 7
3.1. Transforms and spectra 7
3.2. Wave luminosity saturation at high Reynolds number 8
3.3. Wave luminosity of different stars 9

4. The Transfer Function: Connecting Stellar Variability to Convective Driving 10

5. Verification of the Transfer Function in Wave Propagation Simulations 12
5.1. Surface perturbations of standing gravity waves 12
5.2. Calculation of the transfer function for the Wave Propagation simulation 12
5.2.1. Timestepping errors in high-frequency waves and Wave Propagation simulation transfer functions 13
5.3. Calibrating $A_{\rm corr}$ and comparing Wave Propagation data with transfer function solution 15

6. Photometric Variability Spectra 16
6.1. The transfer function 16
6.2. Synthesis of photometric variability spectra 17
6.3. Red noise fits to synthesized spectra 19

7. The Effects of Rotation 19
7.1. Observations of stellar rotation and red noise 19
7.2. A $15M_\odot$ simulation with $P_{\rm rot} = 10$ d 20

1. MESA STELLAR MODELS

1.1. *Near-ZAMS calculations used in this work*

Our 1D stellar evolution calculations were performed using the Modules for Experiments in Stellar Astrophysics software instrument (Paxton et al. 2011, 2013, 2015, 2018, 2019; Jermyn et al. 2022b, MESA `r21.12.1`). We evolve stars from the pre-main sequence to a state near the zero-age main sequence (ZAMS) using the `stop_near_zams` flag and setting `Lnuc_div_L_zams_limit` = 0.99. Input mass and metallicity and output surface properties of these ZAMS stars are shown in Supplementary Table 1. Full MESA inlists and the stellar profiles that were used in this work are available online in the Github repository https://github.com/evanhanders/gmode_variability_paper.

In section 7 of these supplemental materials, we retrieve stellar luminosity, temperature, and convective timescale data from main sequence models presented in Jermyn et al. (2022a); the models in that paper only extend up to a maximum mass of $60M_\odot$, so we additionally run $70-120\ M_\odot$ stellar models using the inlist template from that work.

1.2. *Input physics*

The MESA EOS is a blend of the OPAL (Rogers & Nayfonov 2002), SCVH (Saumon et al. 1995), FreeEOS (Irwin 2004), HELM (Timmes & Swesty 2000), PC (Potekhin & Chabrier 2010), and Skye (Jermyn et al. 2021) EOSes.

Radiative opacities are primarily from OPAL (Iglesias & Rogers 1993, 1996), with low-temperature data from Ferguson et al. (2005) and the high-temperature, Compton-scattering dominated regime by Poutanen (2017). Electron conduction opacities are from Cassisi et al. (2007) and Blouin et al. (2020).

Nuclear reaction rates are from JINA REACLIB (Cyburt et al. 2010), NACRE (Angulo et al. 1999) and additional tabulated weak reaction rates Fuller et al. (1985); Oda et al. (1994); Langanke & Martínez-Pinedo (2000). Screening is included via the prescription of Chugunov et al. (2007). Thermal neutrino loss rates are from Itoh et al. (1996).

Supplementary Table 1. Properties of the stellar models studied in this work. Input (mass M, metallicity Z) and output surface properties (stellar radius R_*, luminosity L, effective temperature $T_{\rm eff}$, surface gravity g and spectroscopic luminosity $\mathscr{L} = T_{\rm eff}^4/g$). We take $R_\odot = 6.957 \times 10^{10}$ cm, $L_\odot = 3.828 \times 10^{33}$ erg/s, $g_\odot = 2.7 \times 10^4$ cm s^{-2}, and $\mathscr{L}_\odot = 4.06 \times 10^{10}$ K^4 s^2/cm.

M ($M_\odot$)	Z	R_* ($R_\odot$)	$\log_{10}(L/L_\odot)$	$\log_{10}(T_{\rm eff}/{\rm K})$	$\log_{10}(g/g_\odot)$	$\log_{10}(\mathscr{L}/\mathscr{L}_\odot)$
3	0.014	1.921	1.970	4.112	-0.089	1.492
15	0.006	4.289	4.289	4.517	-0.088	3.111
40	0.014	8.177	5.359	4.645	-0.223	3.755

2. DEDALUS EQUATIONS & SIMULATION DETAILS

2.1. *Equation formulation*

The simulations presented in this work time-evolve the fully compressible Navier-Stokes equations (Landau & Lifshitz 1987, §15 and §49). We close these equations using the equation of state of a simple perfect gas (Weiss et al. 2004, §9.15), $P = (\mathcal{R}/\mu)\rho T$, where P is the pressure, ρ is the mass density, T is the temperature, $\mathcal{R}$ is the universal gas constant, and μ is the mean molecular weight which we take to be constant.

Dedalus uses implicit-explicit timestepping techniques, where the linear terms (thus linear waves) can be timestepped implicitly, while the nonlinear terms can be timestepped explicitly. To take advantage of this, we decompose our thermodynamics into background components (subscript 0) and fluctuating components (subscript 1) as

$$\ln\rho = (\ln\rho)_0 + (\ln\rho)_1, \qquad s = s_0 + s_1, \qquad \varpi = \varpi_0 + \varpi_1 + \varpi_2, \tag{1}$$

where s is the specific entropy and we have defined a modified temperature

$$\varpi \equiv \frac{\mathcal{R}}{\mu}T. \tag{2}$$

We assume that the background state is in hydrostatic equilibrium. We evaluate the linear and nonlinear fluctuations ϖ_1 and ϖ_2 from the equation of state (c.f., Eqn. 9.39 of Weiss et al. 2004),

$$\frac{\varpi_1}{\varpi_0} = \gamma\left(\frac{s_1}{c_p} + \frac{\gamma-1}{\gamma}[\ln\rho]_1\right), \qquad \text{and} \qquad \frac{\varpi_2}{\varpi_0} \equiv \exp\left(\frac{\varpi_1}{\varpi_0}\right) - \left(1 + \frac{\varpi_1}{\varpi_0}\right), \tag{3}$$

so $T_1 = \varpi_1/(\mathcal{R}/\mu)$ and $T_2 = \varpi_2/(\mathcal{R}/\mu)$. Note also that $(\varpi_1/\varpi_0) = \ln T - \ln T_0$.

Under this thermodynamic decomposition, the fully compressible equations can be written with the implicit terms on the LHS of the equations and the explicit terms on the RHS of the equations,

$$\partial_t \ln\rho_1 + \nabla\cdot\mathbf{u} + \mathbf{u}\cdot\nabla[\ln\rho]_0 = -\mathbf{u}\cdot\nabla[\ln\rho]_1 \tag{4}$$

$$\begin{aligned}\partial_t\mathbf{u} + \gamma\varpi_0\left(\frac{\nabla s_1}{c_p} + \nabla[\ln\rho]_1\right) + \mathbf{g}\frac{\varpi_1}{\varpi_0} - [\nu(\nabla\cdot\bar{\sigma} + \bar{\sigma}\cdot\nabla(\ln\rho)_0) + \bar{\sigma}\cdot\nabla\nu] + \mathcal{D}(r)\mathbf{u} \\ = -\mathbf{u}\cdot\nabla\mathbf{u} - 2\mathbf{\Omega}\times\mathbf{u} - \left[\gamma\varpi'\left(\frac{\nabla s_1}{c_p} + \nabla[\ln\rho]_1\right) + \mathbf{g}\frac{\varpi_2}{\varpi_0}\right] + \nu\bar{\sigma}\cdot\nabla(\ln\rho)_1,\end{aligned} \tag{5}$$

$$\partial_t s_1 + \mathbf{u}\cdot\nabla s_0 + \left(\frac{1}{\rho_0 T_0}\right)[\nabla\cdot(\mathbf{F}_{\rm rad})] = -\mathbf{u}\cdot\nabla s_1 + \left(\frac{1}{\rho T} - \frac{1}{\rho_0 T_0}\right)[\nabla\cdot(-\mathbf{F}_{\rm rad})] + \frac{1}{\rho T}[\Phi + Q], \tag{6}$$

where $\mathbf{u}$ is the velocity, $\mathbf{g}$ is the gravitational acceleration, $F_{\rm rad}$ is the radiative flux, Q is internal heating and cooling from nuclear burning and radiative flux divergences, ν is the kinematic viscosity, γ is the adiabatic index, and c_p is the specific heat at constant pressure. We divide the standard viscous stress tensor by $\rho\nu$ to define

$$\sigma_{ij} = 2\left(E_{ij} - \frac{1}{3}\delta_{ij}\nabla\cdot\mathbf{u}\right), \qquad \text{with} \qquad \bar{\mathbf{E}} = \frac{1}{2}\left(\nabla\mathbf{u} + [\nabla\mathbf{u}]^T\right). \tag{7}$$

The viscous heating term is

$$\Phi = 2\rho\nu\left[\mathrm{Tr}(\bar{\mathbf{E}}\cdot\bar{\mathbf{E}}) - \frac{1}{3}(\nabla\cdot\mathbf{u})^2\right] \tag{8}$$

where Tr is the trace operation. While some authors neglect Φ because viscosity is expected to be small in stellar interiors, this term is required for energy conservation and the "zeroth law of turbulence" (Pope 2000) states that the size of the dissipation term is set by the (large) scale at which energy is injected into the turbulent cascade, not the (small) dissipation scale. Interestingly, Currie & Browning (2017) found that decreasing the viscosity can *increase* the size of viscous dissipation, so this term is crucial to include.

Here, the term with $\mathcal{D}(r)$ is a damping term (see Sec. 2.5), and we study one rotating simulation in Sec. 7, so we include the Coriolis term $2\mathbf{\Omega}\times\mathbf{u}$, where $\mathbf{\Omega} = \hat{z}(2\pi/P_{\rm rot})$ and $P_{\rm rot}$ is the rotation period of the star, and we set $\mathbf{\Omega} = \mathbf{0}$ for nonrotating runs. As we detail in Sec. 2.6, the heating term Q in Eqn. 6 includes both nuclear burning and the divergence of the radiative flux from the MESA model. As a result, we only solve for the radiative flux associated with the temperature fluctuations around the background state.

We further simplify the radiative flux term by neglecting the fully nonlinear portion of the radiative flux associated with T_2 and only solve for the flux associated with the linear perturbations to the temperature, $\mathbf{F}_{\rm rad} = -k_{\rm rad}\nabla T_1 = -k_{\rm rad}\nabla(\varpi_1/[\mathcal{R}/\mu])$. The viscous diffusivities and radiative conductivities used in our simulations are specified in Sec. 2.6. The characteristic Mach numbers of of convective flows are very low (see Supplementary Table 3), and we expect linear temperature perturbations of the order $T_1/T_0 \sim \mathrm{Ma}^2 \sim 10^{-6}$ (Anders & Brown 2017), and we expect nonlinear terms to be of order $\mathrm{Ma}^4 \sim 10^{-12}$ per Eqn. 3 (because $\varpi_2/\varpi_0 \approx \mathcal{O}([\varpi_1/\varpi_0]^2)$ after Taylor expanding the exponential), so this term that we neglect is small. Aside from the inclusion of the Coriolis term in a rotating frame and linearizing the radiative flux, Eqns. 4-6 are a mathematically-equivalent restatement of the fully compressible equations.

2.2. *Boundary conditions*

At the outer boundary $r = R$ of the `Dedalus` simulation, we impose the following boundary conditions:

$$\hat{e}_r\cdot\nabla\varpi_1(r=R) = 0, \qquad \hat{e}_r\cdot\mathbf{u}(r=R) = 0, \qquad E_{r,\phi}(r=R) = E_{r,\theta}(r=R) = 0. \tag{9}$$

These conditions make it so that there are no flux, velocity, or angular stress perturbations at the outer boundary of the simulation. We split the simulation into different radial domains, and expand all variables in terms of different

Supplementary Table 2. Characteristic scales used in simulations. Input (mass M, metallicity Z) parameters of the MESA models and associated nondimensional quantities used in Wave Generation (WG) and Wave Propagation (WP) Dedalus simulations, as well as the characteristic heating timescale, the MLT velocity, and the value of the specific heat at constant pressure (c_p) and adiabatic index (γ) used in the simulation.

M ($M_\odot$)	Z	Sim. Type	L_C (cm)	m_C (g)	T_C (K)	τ_nd (s)	τ_heat (s)	$\langle u_\mathrm{MLT}\rangle$ (cm s^{-1})	c_p (erg g^{-1}K^{-1})	γ
3	0.014	WG	1.87×10^{10}	1.93×10^{32}	1.89×10^{7}	3.25×10^{3}	5.74×10^{5}	1.37×10^{4}	3.49×10^{8}	1.654
15	0.006	WG	8.21×10^{10}	1.89×10^{33}	2.35×10^{7}	8.41×10^{3}	4.66×10^{5}	6.68×10^{4}	3.88×10^{8}	1.560
15	0.006	WP	8.21×10^{10}	1.89×10^{33}	2.35×10^{7}	6.09×10^{3}	4.66×10^{5}	6.68×10^{4}	3.88×10^{8}	1.560
40	0.014	WG	2.06×10^{11}	6.01×10^{33}	2.11×10^{7}	1.52×10^{4}	5.55×10^{5}	1.33×10^{5}	4.20×10^{8}	1.481

basis functions across each radial domain (see Sec. 2.4). At the boundaries of the radial domains, we impose continuity of $\hat{e}_r \cdot \mathbf{u}$, s_1, $(\ln\rho)_1$, $\hat{e}_r \cdot \nabla(\varpi_1/\varpi_0)$, $E_{r,\phi}$, and $E_{r,\theta}$. For the $\ell = 0$ mode, instead of specifying that $\hat{e}_r \cdot \nabla\varpi_1(r = R) = 0$, we instead specify that there are no fluctuations in the volume-integrated total system energy $\mathcal{E} = 0.5\rho|u|^2 + \rho\phi_g + P/(\gamma-1)$ (where ϕ_g is the gravitational potential); while these are mathematically equivalent conditions, they can in practice produce slightly different solutions due to the accumulation of timestepping errors from e.g., the tau-method enforcement of boundary conditions over many timesteps[1].

2.3. *Nondimensionalization*

We take the radius of the core convection zone of our star to be our nondimensional unit of length, $L_\mathrm{C} = r_\mathrm{core}$. We use the density and temperature values at that radial coordinate to set the nondimensional temperature and mass units, $T_\mathrm{C} = T(r = r_\mathrm{core})$ & $m_\mathrm{C} = \rho(r_\mathrm{core})L_\mathrm{C}^3$. We use the maximum value of the buoyancy frequency in our simulation domain to determine the nondimensional time unit, $\tau_\mathrm{nd} = (2\pi)/\max(N)$. From these four fundamental units, all other quantities (energy, entropy, etc.) can be constructed.

We find that convective dynamics occur on a timescale which is related to the nuclear burning rate in the core, $\tau_\mathrm{heat} = [\epsilon(r = 0)/L_\mathrm{C}^2]^{-1/3}$, where ϵ is the energy generation rate in erg g^{-1} s^{-1}, and we evolve our simulations for tens to hundreds of heating timescales. Note that τ_heat is closely related to a standard mixing length velocity timescale $\tau_\mathrm{mlt} = (\rho/F_\mathrm{conv})^{1/3}$, under the relation $F_\mathrm{conv} \sim \rho\epsilon L_\mathrm{C}$, which is a decent approximation. We refer the reader to Supplementary Table 2 for key nondimensional values. To implement the equations in nondimensional form, we implement the equations as written in Eqns. 4-6, but we multiply all quantities in the equations by the proper dimensional scales from Supplementary Table 2 to create dimensionless quantities, so that e.g., the simulation velocity $u_\mathrm{sim} = (\tau_\mathrm{nd}/L_\mathrm{C})u_\mathrm{dimensional}$. This is equivalent to multiplying the log-density Eqn. 4 by τ_nd, the velocity Eqn. 5 by $\tau_\mathrm{nd}^2/L_\mathrm{C}$, and the entropy Eqn. 6 by $\tau_\mathrm{nd}/s_\mathrm{nd}$ where $s_\mathrm{nd} = L_\mathrm{C}^2/\tau_\mathrm{nd}^2/T_\mathrm{C}$

2.4. *Discretization and timestepping choices*

We solve Eqns. 4-6 along with the boundary conditions in Section 2.2 using version 3 of the Dedalus pseudospectral framework (Burns et al. 2020). Simulations scripts can be found in the `gmode_variability_paper`[2] Github repository; these scripts rely on the separate `compressible_stars`[3] Github repository and were run using the `master` branch of the `Dedalus` Github repository[4] at the commit with short-sha `29f3a59`. The simulation domain is decomposed into a `BallBasis` (with nr_B radial coefficients) and then either one (for Wave Generation simulations) or two (for the Wave Propagation simulation) `ShellBasis` objects (with nr_{s1}, nr_{s2} radial coefficients). In the main text, the "number of resolution elements across the convective core" is $2\,\mathrm{nr}_B$, which is the number of elements across the *diameter* of the `BallBasis` for comparison with "star in a box" simulations performed in Cartesian domains. The `BallBasis` expands variables using a basis of radially-weighted Zernike polynomials (Vasil et al. (2019), though see section 6.1.2 of Lecoanet et al. (2019) for subtleties of resolution) and the `ShellBasis` expands variables radially using a Chebyshev

[1] We find that enforcing energy conservation in this way introduces a small stochastic forcing into $\ell = 0$ sound waves. There are no $\ell = 0$ gravity waves , so we do not analyze the $\ell = 0$ motions in this paper. We have verified that using a standard gradient boundary condition for $\ell = 0$ (e.g., $\hat{e}_r \cdot \nabla\varpi_1 = 0$) does not affect the power of gravity waves.

[2] https://github.com/evanhanders/gmode_variability_paper

[3] https://github.com/evanhanders/compressible_stars

[4] https://github.com/DedalusProject/dedalus

polynomial basis. The `BallBasis` radially spans $r \in [0, 1.1]$ in nondimensional units. In Wave Generation simulations, the `ShellBasis` spans $r \in [1.1, 2]$, and for Wave Propagation simulations, the two `ShellBasis` domains respectively span $r \in [1.1, 2.98]$ and $r \in [2.98, 3.38]$. Variables are expanded angularly over azimuth $\phi \in [0, 2\pi]$ and colatitude $\theta \in [0, \pi]$ using spin-weighted spherical harmonics with maximum harmonic degree $L_{\rm max}$ and angular wavenumbers $m \in [-L_{\rm max}, L_{\rm max}]$.

Initial value problems ("simulations") use the time-stepper SBDF2 (Wang & Ruuth 2008) with a CFL safety factor of 0.2. For each background state, we initialize a high-diffusivity simulation using noise in the entropy field of order 10^{-8}. To achieve more turbulent simulations, we "bootstrap" simulations by using the nonlinear dynamical state of a lower resolution simulation as initial conditions and increasing the resolution while decreasing the diffusivity. This technique avoids the transient associated with the onset of the convective instability from a stationary state.

Spectral methods with finite coefficient expansions cannot capture true discontinuities. To approximate discontinuous functions, we define a smooth Heaviside step function centered at $r = r_0$,

$$H(r; r_0, d_w) = \frac{1}{2}\left(1 + \mathrm{erf}\left[\frac{r - r_0}{d_w}\right]\right). \tag{10}$$

where erf is the error function and d_w determines the width of the smooth transition.

2.5. *Damping layers*

Wave Generation simulations include a damping layer in Eqn. 5 of the form,

$$\mathcal{D}(r) = \tau_{\rm D}^{-1}[1 - H(r; 1.85, 0.07)], \tag{11}$$

which introduces a Newtonian damping to the velocity field in the outer ∼15-20% of the radiative zone, similar to the damping layer introduced by Couston et al. (2018) and Lecoanet et al. (2021). In Wave Generation simulations, we take $\tau_{\rm D}^{-1} = \tau_{\rm nd}^{-1}$, the timescale associated with the maximum buoyancy frequency in the simulation domain. In Wave Propagation simulations, we set $\mathcal{D}(r) = 0$.

2.6. *Constructing the background state from MESA models*

The background state in our simulations is constructed using information from MESA stellar models. We take the γ and c_p to be constants and set them to their values at $r = 0$ in the MESA model.

In the `BallBasis`, $r \leq 1.1$, it is crucial for the background fields to satisfy the regularity of the polynomial basis as $r \to 0$. To begin constructing our simulation structure, we fit a quadratic polynomial $\tilde{N}^2 = A + Br^2$ such that $\tilde{N}^2(r = 1.1) = N^2_{\rm MESA}(r = 1.1)$ and $\nabla\tilde{N}^2(r = 1.1) = \nabla N^2_{\rm mesa}(r = 1.1)$, and we construct the simulation N^2 profile as

$$N^2_{\rm sim} = \begin{cases} H(r; 1.07, 0.03)\tilde{N}^2 & r \leq 1.1 \\ N^2_{\rm MESA} & r > 1.1 \end{cases}, \tag{12}$$

where H is defined in Eqn. 10. We next define the simulation convective luminosity $L_{\rm conv,sim}$. The MESA convective luminosity $L_{\rm conv,MESA}$ is discontinuous at the outer edge of the convective core (at $r = 1$), so we use Eqn. 10 to make it smoothly transition to zero at the convective core boundary and use that smoothed version as $L_{\rm conv,sim}$. We then solve for the simulation heating term by evaluating $Q_{\rm sim} = \nabla\cdot F_{\rm conv,sim}$, where $F_{\rm conv,sim} = L_{\rm conv,sim}/(4\pi r^2)$. Note that since $Q_{\rm sim}$ is based on the divergence of the *convective* flux rather than the total stellar flux or luminosity, it is encoded both with heating information from nuclear reactions and cooling information associated with changing radiative luminosity.

We set the simulation density profile $\ln\rho_{0,\rm sim} = \ln\rho_{\rm MESA}$. We construct a hydrostatically-equilibrated background state with the specified $N^2_{\rm sim}$ and $\ln\rho_{0,\rm sim}$ by using `Dedalus` to solve the following boundary value problem for s_0 and ϖ_0 on our spectral bases,

$$\nabla\ln\rho_{0,sim}\cdot\nabla\left(\frac{s_{0,sim}}{c_p}\right) = -\frac{N^2_{\rm sim}}{\gamma\varpi_{0,sim}} - \frac{|\nabla s_{0,sim}|^2}{c_p^2}, \text{ with } \varpi_0 = \frac{\mathcal{R}}{\mu}\exp\left(\gamma\left[\frac{s_{0,sim}}{c_p} + \frac{\gamma-1}{\gamma}\ln\rho_{0,sim}\right]\right), \tag{13}$$

under the boundary conditions that $\ln(\varpi_{0,sim})(r = 1) = 0$ and $s_{0,\rm sim}$ is continuous at the interfaces of different spectral bases. Once we know the thermal stratification, we solve for the gravitational potential, ϕ_g,,

$$\nabla\phi_{g,\rm sim} - \gamma\varpi_{0,\rm sim}\left(\frac{\nabla s_{0,sim}}{c_p} + \nabla\ln\rho_{0,\rm sim}\right) = 0 \tag{14}$$

while specifying that $\phi_{g,\mathrm{sim}}(r = R) = 0$ (where R is the outermost radial coordinate of the simulation domain), and $\phi_{g,\mathrm{sim}}$ is continuous at the interfaces of different spectral bases. Eqn. 13 solves for the hydrostatically equilibrated s_0 under the constraints of the specified N^2 and $\ln \rho_0$ profiles.

We specify the viscous diffusivity ν_{sim} present in our simulations to control the degree of turbulence. To construct the radiative diffusivity profile used in our simulations, we set $\chi_{\mathrm{rad}} = \nu_{\mathrm{sim}} + \chi_{\mathrm{MESA}}$ where $\chi_{\mathrm{MESA}} = 16\sigma_{\mathrm{SB}}T^3/(3\rho^2 c_p \kappa)$, where σ_{SB} is the Stefan-Boltzmann constant, and κ is the opacity. We then use this radial profile to construct the simulation radiative conductivity $k_{\mathrm{rad}} = \rho_{\mathrm{sim}} c_p \chi_{\mathrm{rad}}$. In the convective core, ν_{sim} is larger than the viscous diffusivity and radiative diffusivity present in the MESA model. In the outer envelope of our 15 $M_\odot$ Wave Propagation simulation, the radiative diffusivity in the simulation and MESA model become larger than ν_{sim} (see Supplementary Fig. 1). The Prandtl number $\mathrm{Pr} = \nu/\chi_{\mathrm{rad}} = 1$ in the convection zone and $\mathrm{Pr} < 1$ near the surface of the Wave Propagation simulation.

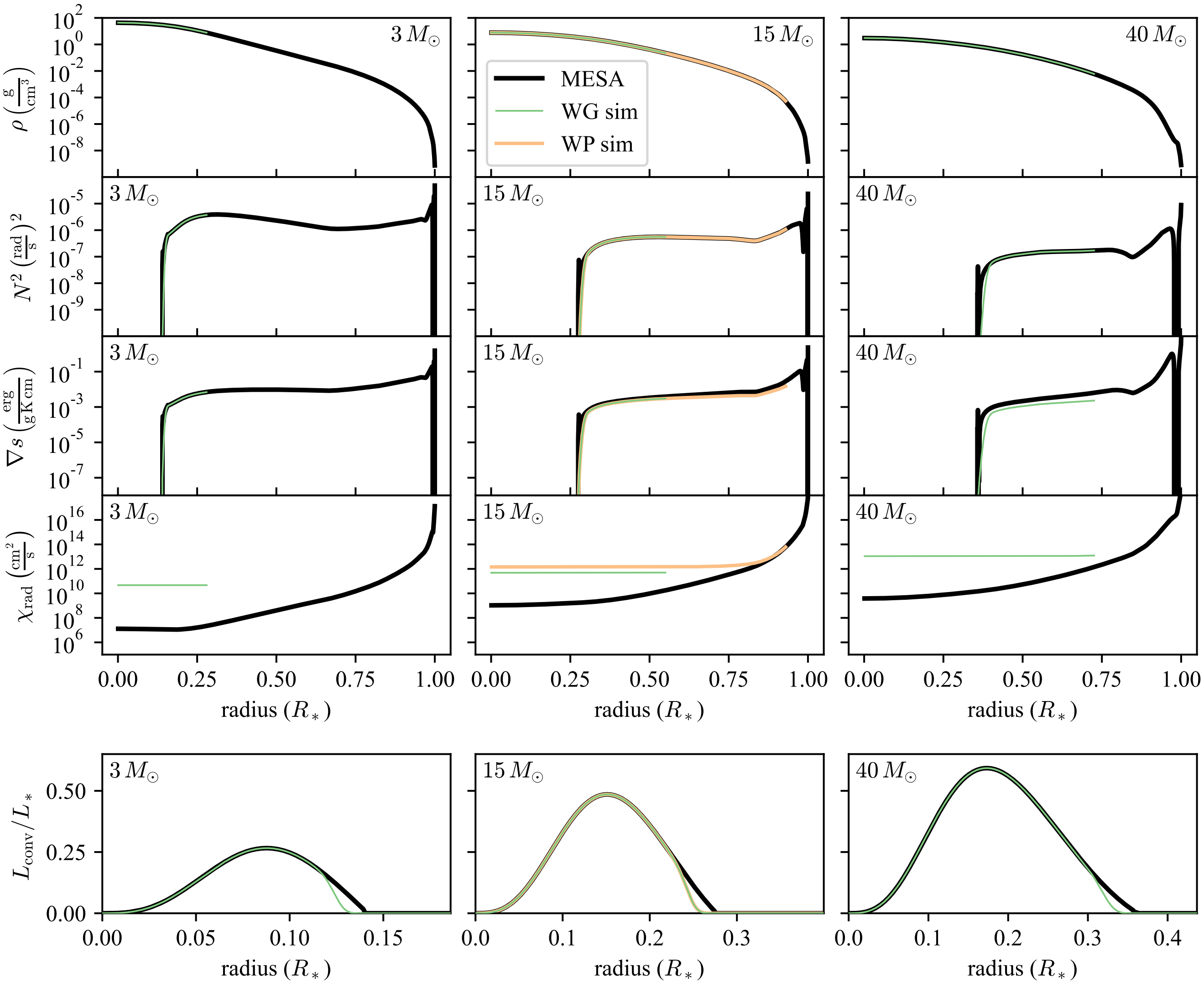

Supplementary Figure 1. Comparison between MESA stellar models and Dedalus simulation profiles. Radial profiles of various quantities from MESA stellar models are shown in black for the 3 $M_\odot$ (left), 15 $M_\odot$ (middle), and 40 $M_\odot$ (right) stars; these profiles are compared to Wave Generation simulations (WG, green) and the Wave Propagation simulation (WP, peach). We show the density ρ (top row), the square of the Brunt-Väisälä frequency (second row), the specific entropy gradient (third row), and the radiative diffusivity (fourth row). In the bottom row, we zoom in the x-axis on the core convection zone and show the convective luminosity.

Comparisons between profiles of ρ, N^2, ∇s, $\chi_{\rm rad}$, and the convective luminosity $L_{\rm conv}$ in the MESA model and our Dedalus simulations are shown in Supplementary Fig. 1. Full MESA profiles are shown as black lines, while Wave Generation (WG) simulations are shown as green lines and our Wave Propagation (WP) simulation is shown as a peach colored line. As expected, our ρ and N^2 profiles faithfully reproduce the MESA model, except right above the core convection zone where we intentionally smooth the MESA profile (Eqn. 12). In the 3 $M_\odot$ star, ∇s also faithfully reproduces the MESA model, but for higher mass stars there is a slight disagreement between the MESA and Dedalus ∇s profiles. This occurs because our equation of state does not capture radiation pressure which becomes important in higher mass stars, and it should be included in future studies of the highest mass stars. $L_{\rm conv}$ reproduces the stellar convective luminosity, except at the outside of the convection zone where we explicitly smoothed the heating profile.

2.7. *Table of simulations*

Resolution and input characteristics and key output quantities from the simulations conducted in this work are shown in Supplementary Table 3. The 15 $M_\odot$ model is our fiducial model. The two simulations displayed in Fig. 1 of the main text are the 15 $M_\odot$ WP simulation and the highest-resolution 15 $M_\odot$ WG simulation ($L_{\rm max}+1 = 512$). The predictions presented in Fig. 2 of the main text use the Wave Luminosity measured from the three WG simulations with $L_{\rm max+1} = 256$ for the 3, 15, and 40 $M_\odot$ models.

Supplementary Table 3. Input and basic output values from the Dedalus simulations in this work. Columns show the star model mass, simulation type (WP - Wave Propagation or WG - Wave Generation), the number of spherical harmonic degrees used (including $\ell = 0$), the number of coefficients in the radial coefficient expansion ($\{{\rm nr}_B,\ {\rm nr}_{s1},\ {\rm nr}_{s2}\}$ for WP, $\{{\rm nr}_B,\ {\rm nr}_{s1}\}$ for WG), the viscous diffusivity in the simulation, the stellar rotation period in days, the simulation run time in heating timescales, and the core-average of the velocity, Mach number, and Reynolds number (Re $= \langle u\rangle_{\rm core} L_{\rm C}/\nu_{\rm sim}$). The heating timescales are shown in Supplementary Table 2.

Model Star	Sim. Type	$L_{\rm max}+1$	$N_{\rm max}+1$	$\nu_{\rm sim}$ (cm^2 s^{-1})	$P_{\rm rot}$ (d)	$t_{\rm sim}$ ($t_{\rm heat}$)	$\langle u\rangle_{\rm core}$ (cm s^{-1})	$\langle {\rm Ma}\rangle_{\rm core}$	$\langle {\rm Re}\rangle_{\rm core}$
15	WP	256	{256,192,64}	1.44×10^{12}	∞	324	5.77×10^{4}	9.17×10^{-4}	3.28×10^{3}
15	WG	512	{512,192}	4.81×10^{11}	∞	39	5.85×10^{4}	9.29×10^{-4}	9.97×10^{3}
15	WG	384	{384,192}	7.22×10^{11}	∞	43	5.84×10^{4}	9.29×10^{-4}	6.64×10^{3}
15	WG	256	{256,128}	1.44×10^{12}	∞	100	5.72×10^{4}	9.08×10^{-4}	3.25×10^{3}
15	WG	256	{256,128}	1.44×10^{12}	10	120	7.94×10^{4}	1.26×10^{-3}	4.51×10^{3}
15	WG	128	{128,96}	3.60×10^{12}	∞	100	5.40×10^{4}	8.57×10^{-4}	1.23×10^{3}
15	WG	128	{128,96}	7.22×10^{12}	∞	100	5.05×10^{4}	8.01×10^{-4}	5.74×10^{2}
15	WG	96	{96,64}	1.44×10^{13}	∞	100	4.62×10^{4}	7.32×10^{-4}	2.63×10^{2}
3	WG	256	{256,128}	4.69×10^{10}	∞	50	9.00×10^{3}	1.68×10^{-4}	3.04×10^{3}
40	WG	256	{256,128}	9.83×10^{12}	∞	35	1.31×10^{5}	2.10×10^{-3}	2.47×10^{3}

3. THE WAVE LUMINOSITY SPECTRUM IN WAVE GENERATION SIMULATIONS

3.1. *Transforms and spectra*

All wave luminosity and power spectra in this work are calculated from long time series of data projected onto a 2D sphere. Given a 3D data cube of a variable $a(t, \phi, \theta)$, we transform the data into frequency space as

$$a(t,\phi,\theta) \underset{\rm SHT}{\longrightarrow} a_{\ell,m}(t) \underset{\rm FT}{\longrightarrow} \hat{a}_{\ell,m}(f). \tag{15}$$

We perform the spherical harmonic transform (SHT) using Dedalus. Dedalus returns two coefficient amplitudes, b_1 and b_2, corresponding to the $\cos(m\phi)$ and $-\sin(m\phi)$ components. From these, we construct the spherical harmonic amplitude $a_{\ell,m}(t) = b_1 + ib_2$, and we normalize the coefficients so that $(1/4\pi)\int_\phi\int_\theta a^2 \sin\theta d\theta d\phi = \sum_{\ell,m} a^*_{\ell,m} a_{\ell,m}$; this ensures that our coefficients are normalized in the same way as SciPy's `sph_harm` function.

We calculate the Discrete Fourier Transform using NumPy and define its normalization such that

$$\hat{a}_{\ell,m,f} = \frac{1}{N}\sqrt{\frac{8}{3}}\sum_{j=0}^{N-1} H_N(j)\hat{a}_{\ell,m}(t_j)\exp\left\{-2\pi i\frac{jf}{N}\right\} \tag{16}$$

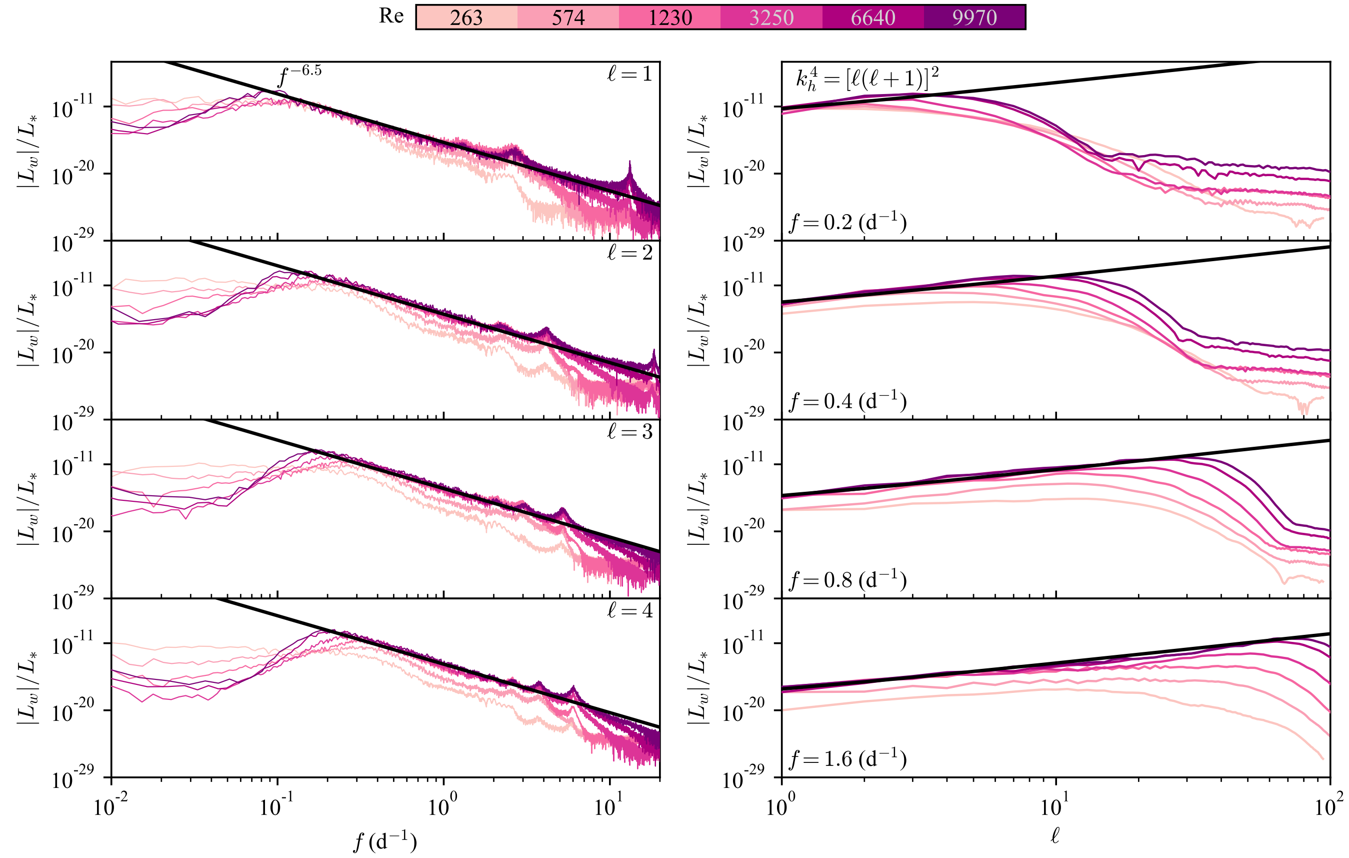

Supplementary Figure 2. Convergence of the wave luminosity as flows become more turbulent. We plot the absolute value of the wave luminosity normalized by the stellar luminosity in Wave Generation simulations of our 15 $M_\odot$ model. (Left panels) Shown is the wave luminosity spectrum vs. frequency at $\ell = [1, 2, 3, 4]$ (from top to bottom). (Right panels) Shown is the wave luminosity spectrum vs. ℓ at $f = [0.2, 0.4, 0.8, 1.6]$ d^{-1} (from top to bottom). Lines are colored by the Reynolds number of the simulation (see Supplementary Table. 3), with the most turbulent simulation being the darkest line. The black line shows the fit from Eqn. 20 for the 15 $M_\odot$ star.

In this definition, $H_N(j)$ is the jth point of the Hanning window defined over N total data points, and the factor of $\sqrt{8/3}$ accounts for this window.

To calculate a power-like quantity (power spectrum, wave luminosity spectrum), we multiply one transformed field with the complex conjugate of another, e.g., $P_A(f) = \hat{a}^* \, \hat{a}$. To properly account for power in negative frequencies, we define $P_A(f) = P_A(f) + P_A(-f)$ for all $f \geq 0$.

Data are measured at 30 minute intervals of simulation time, matching the observational "long-cadence" observing mode of the K2 (Howell et al. 2014) and TESS (Ricker et al. 2014) satellites.

3.2. *Wave luminosity saturation at high Reynolds number*

We perform many Wave Generation simulations of the 15 $M_\odot$ model where we simultaneously increase the spatial resolution while decreasing the diffusivity, see Supplementary Table 3. We measure the wave luminosity spectrum at $r = 1.25$,

$$L_w(f, \ell) = \sum_m 4\pi r^2 \hat{H}_{\ell,m}(f)^* \hat{u}_{r,\ell,m}(f), \tag{17}$$

where the enthalpy is $H = \rho h = (c_p \mu/\mathcal{R})P$ and the radial velocity is $u_r = \mathbf{u} \cdot \hat{e}_r$. We plot the wave luminosity at $\ell = [1, 2, 3, 4]$ and $f = [0.2, 0.4, 0.8, 1.6]$ d^{-1} in Supplementary Fig. 2. We find a wave luminosity spectrum that can be fit to a power-law $L_w \propto f^{-13/2} k_h^4$, with $k_h = \sqrt{\ell(\ell+1)}$. Although we only plot a few values of ℓ and f, we verified that these power-laws work well for many other values, and the full wave luminosity data cubes are available online in a Zenodo repository (Anders et al. 2023). This wave luminosity spectrum was theoretically predicted by Lecoanet &

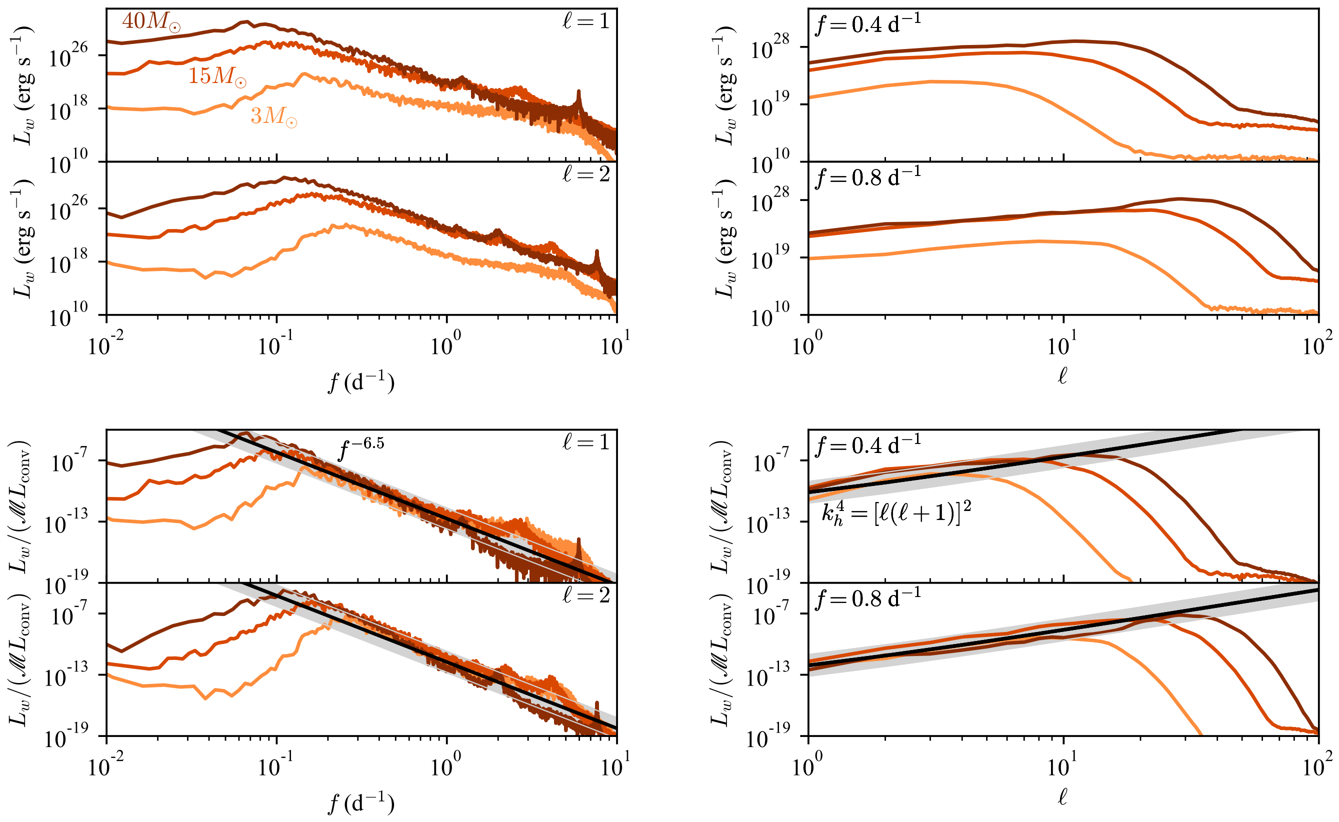

Supplementary Figure 3. The wave luminosity follows a universal power law even for stars of different masses. We plot the wave luminosity spectra from Wave Generation simulations with 512 resolution elements across the convection zone for the 3 $M_\odot$ (light orange), 15 $M_\odot$ (dark orange), and 40 $M_\odot$ (brown) models. As in Supplementary Fig. 2, we plot the spectra vs. frequency in the left panel at $\ell = [1, 2]$ and vs. ℓ in the right panel at $f = [0.4, 0.8]$ d^{-1}. In the top four panels, we plot the wave luminosity in cgs units. In the bottom four panels, we divide by $\mathcal{M}L_{\rm conv}$, where $\mathcal{M}$ is the characteristic convective Mach number of core convection and $L_{\rm conv}$ is the core convective luminosity. The black line shows the fit quoted in Eqn. 18, and the grey band spans an order of magnitude above and below this fit line.

Quataert (2013) and observed in simulations run using the `Dedalus` (Couston et al. 2018; Lecoanet et al. 2021) and `MUSIC` codes (Le Saux et al. 2022, 2023), but is very different from the plume-based wave energy spectrum observed by Rogers et al. (2013). As the diffusion lowers and turbulence increases, we find that the wave luminosity increases at frequencies $f \gtrsim 0.3$ d^{-1}. Once the diffusivities are decreased enough to reach Re $\gtrsim 2000$ (requiring 512 degrees of freedom across the CZ), the wave luminosity ceases to increase as the diffusivities are further decreased. We therefore conclude that the wave luminosity is numerically converged once there are 512 degrees of freedom across the CZ, and we run our Wave Generation simulations for the 3 and 40 $M_\odot$ models with this grid resolution.

We do note that further decreasing the diffusivity changes the range of the frequencies (at low frequency) and spherical harmonic degrees (high ℓ) that are filled by the powerlaw wave luminosity spectrum. In a star, we would therefore expect this power law to extend down to very low frequency (to the frequency at which convection drives the waves), and up to very high spherical harmonic degree. Note also that the wave luminosity spectrum includes small resonant peaks, such as the one around 1.5 d^{-1}. These peaks occur because our wave damping layer does not fully damp out these high frequency waves. We have tried increasing and decreasing the radial extent and forcing magnitude of the wave damping layer, and found the settings that we used to work well for damping most waves.

3.3. *Wave luminosity of different stars*

Having verified the resolution required to achieve a reliable measure of the wave luminosity, we now run Wave Generation simulations of stellar models of different masses. We plot the measured wave luminosities in the top panels of Supplementary Fig. 3 for $\ell = [1, 2]$ and $f = [0.4, 0.8]$ d^{-1}. We find similar spectra for all three stars in this work.

The $3M_\odot$ and $15M_\odot$ are well-described by a $L_w \propto f^{-13/2}k_h^4$; the wave luminosity spectrum of the 40 $M_\odot$ star seems to vary more steeply with frequency, perhaps $L_w \propto f^{-15/2}k_h^4$, which has also been seen in simulations performed by Lecoanet et al. (2021) and Le Saux et al. (2022). Regardless, since a spectrum of the form $L_w \propto f^{-13/2}k_h^4$ is theoretically expected, and since this spectrum describes the 3 $M_\odot$ and 15 $M_\odot$ stars well, and also describes the $40M_\odot$ star fairly well below $f \lesssim 0.3$ d^{-1} where the wave luminosity signal is strongest, we will describe the wave luminosity spectrum using $L_w \propto f^{-13/2}k_h^4$.

In the bottom panels of Supplementary Fig. 3, we plot the same wave luminosities, but normalized by $\mathcal{M}L_{\rm conv}$, where $\mathcal{M} = \{1.30, 5.33, 9.48\} \times 10^{-4}$ are the volume-averaged convective mach numbers in the core convection zones for the $\{3, 15, 40\}\,M_\odot$ stars and $L_{\rm conv} = \{5.85 \times 10^{34}, 1.90 \times 10^{37}, 2.28 \times 10^{38}\}$ erg s^{-1} are the convective luminosities of the core convection. The wave luminosities seem to follow a universal powerlaw of the form

$$L_w = (4 \times 10^{-46\pm1})\mathcal{M}L_{\rm conv}\left(\frac{f}{\rm Hz}\right)^{-13/2}(\ell[\ell+1])^2\,, \tag{18}$$

and we plot this line and also the uncertainty range of our exponent as a black line and grayed stripe in Supplementary Fig. 3. We note that theory predicts a general powerlaw of the form (Lecoanet & Quataert 2013; Goldreich & Kumar 1990)

$$L_w \approx \mathcal{M}L_{\rm conv}\left(\frac{f}{f_c}\right)^{-13/2}(\ell[\ell+1])^2\,, \tag{19}$$

where f_c is the characteristic frequency of the core convection. The three stars studied in this work have $f_c = u_{\rm MLT}/R_{\rm core} = \{4.78, 5.30, 3.93\} \times 10^{-7}$ Hz for the $\{3, 15, 40\}\,M_\odot$ stars, so we do not sample enough dynamic range of f_c to characterize that scaling. We fit in log-space the $f^{-13/2}\,(\ell[\ell+1])^2$ spectrum to L_w for each of the stars at $\ell = 1$ and in the range $f = [0.2, 1.5]$ d^{-1} (3 $M_\odot$), $f = [0.2, 1]$ d^{-1} (15 $M_\odot$), and $f = [0.1, 0.7]$ d^{-1} (40 $M_\odot$). These fits are,

$$L_w = \begin{Bmatrix} 7.34 \times 10^{-15}\,{\rm erg\,s^{-1}} & ({\rm for}\,3M_\odot) \\ 2.33 \times 10^{-11}\,{\rm erg\,s^{-1}} & ({\rm for}\,15M_\odot) \\ 5.30 \times 10^{-10}\,{\rm erg\,s^{-1}} & ({\rm for}\,40M_\odot) \end{Bmatrix}\left(\frac{f}{\rm Hz}\right)^{-13/2}(\ell[\ell+1])^2\,, \tag{20}$$

and we will use these when we generate our photometric variability predictions in Section 6.

4. THE TRANSFER FUNCTION: CONNECTING STELLAR VARIABILITY TO CONVECTIVE DRIVING

Appendices A and B of Lecoanet et al. (2019) provide the steps for calculating the transfer function. We carried out a similar calculation following that paper carefully, but with the following changes to the calculation:

1. In Eqns. 12-13 of appendix A of Lecoanet et al. (2019), they introduce a complex oscillatory forcing term proportional to $\exp(i\omega t)$. Later in Eqn. 14, they project out the dual basis, which itself is complex, to determine the real amplitude of each eigenmode. In this projection, imaginary portions of the dual vector can multiply with the imaginary portion of the oscillation to produce unphysical real contributions to the amplitude which are not produced by a purely real forcing process like convection. To fix this, we assume that the forcing is purely real with an arbitrary phase. We furthermore assume that the forcing is purely horizontal, but we do not achieve this by constructing a horizontal gradient of a spherical harmonic as in their Eqn. 13, so we do not include the additional factors of $\sqrt{\ell(\ell+1)}/r$ that come with that gradient. As a result, we replace their appendix A, Eqn. 13 with
$$\mathbf{f}_\ell(t;\omega) = F_{\omega,\ell}\delta(r - r_f)\sin(\omega t + \xi)\hat{\mathbf{e}}_\perp, \tag{21}$$
where $F_{\omega,\ell}$ is the forcing amplitude at angular frequency ω and spherical harmonic degree ℓ, r_f is the forcing radius, δ is the Dirac delta function, ξ is the phase, and $\hat{\mathbf{e}}_\perp$ is a unit vector perpendicular to the gravity.

2. When connecting between the bulk forcing and the interface forcing, we again define the force to be in the horizontal direction but do not use a gradient, which makes our results differ by another factor of $\sqrt{\ell(\ell+1)}/r$. Furthermore, when taking the average response of many bulk delta function forcings (Eqn. 28 in Appendix B of Lecoanet et al. (2019)), we integrate the square $\overline{u}_z^2(z_f)dz_f$, rather than the magnitude, resulting in a slightly different leading constant. As a result, instead of their Eqn. 29, we arrive at
$$F_{\omega,k_h} = \sqrt{2}\frac{\omega}{k_h}F_B. \tag{22}$$

3. We connect the forcing amplitude to the wave luminosity, L_w, defined in Eqn. 17, rather than to the radial velocity of the waves. L_w depends on the enthalpy H and the radial velocity u_r. The enthalpy and radial velocity perturbations are related to each other by the polarization relation. In the limit of low-Mach number flows ($\partial_t \rho \sim 0$) with negligible viscosity, and in the limit where radial derivatives in perturbation variables are small compared to background gradients in the density ($k_r \ll H_\rho^{-1}$ the density scale height)[5], this relationship is
$$H = \mathcal{P} u_r, \qquad \text{with} \qquad \mathcal{P}(N^2; \omega, k_h) = -\rho_0 \frac{c_P \mu}{\mathcal{R}} \frac{\omega}{k_h^2} k_r(N^2, \omega, k_h) \tag{23}$$
Accounting for thermal diffusion instead of viscosity, the dispersion relation for waves under these approximations is given by Eqn. 12 of Lecoanet et al. (2015),
$$\widetilde{k_r} = \frac{(-1)^{3/4}}{\sqrt{2}} \sqrt{-2ik_h^2 - \frac{\omega}{\chi_{\text{rad}}} + \frac{\sqrt{\omega^3 + 4ik_h^2 \chi_{\text{rad}} N^2}}{\chi_{\text{rad}} \sqrt{\omega}}}. \tag{24}$$
The radial wavenumber is the real component $k_r = \Re\{\widetilde{k_r}\}$, and we use k_r in Eqn. 23. Note that k_r is a function of N^2 and thus $\mathcal{P}$ is a function of N^2 and ρ_0, which both depend upon the radius. In evaluating Eqn. 23, we use the maximum N^2 in the inner 93% of the star, which we call N^2_{max}. We make this choice so that we can evaluate the transfer function at high frequency, because otherwise $\widetilde{k_r}$ becomes imaginary at high frequency and gravity waves become evanescent. However, this choice leads to an improper evaluation of the polarization relation $\mathcal{P} \propto k_r$ at low frequency. To correct this, we introduce a factor and define
$$\mathcal{P}' = \frac{N(r_{\text{force}})}{N_{\text{max}}} \mathcal{P}(N_{\text{max}}). \tag{25}$$
We use this relation to express the wave radial velocity in terms of the wave luminosity,
$$|u_r|^2 = \frac{L_w(\omega, \ell)}{4\pi r_{\text{force}}^2 \mathcal{P}'}. \tag{26}$$
Using this, we modify Eqn. 30 of Lecoanet et al. (2019) to set the forcing amplitude with the wave luminosity,
$$F_{\omega, k_h} = \frac{\omega}{k_h} \left(\frac{L_w(\omega, \ell)}{2\pi r_{\text{force}}^2 \mathcal{P}'} \right)^{1/2}. \tag{27}$$

With these changes, we are ready to construct a transfer function for the photometric variability of a star. We assume that the gravity waves which propagate in the envelope of a star or in the radiative zone of our simulation can be described by a set of eigenvalues ω_n and corresponding eigenfunctions describing perturbations to e.g., the photometric magnitude,
$$\Delta m_\ell(R_*, t) = \sum_n A_\ell(t; \omega_n) \widetilde{\Delta m_{\ell,n}}(R_*; \omega_n) \exp\left(-i\omega_n t\right), \tag{28}$$
where the eigenfunctions are denoted by tildes, and $A_\ell(t; \omega_n)$ is the time-dependent amplitude of the wave with eigenvalue ω_n. Here, Δm_ℓ is the photometric magnitude fluctuations at spherical harmonic degree ℓ. In other words, given the eigenfunctions and an expression for the wave amplitudes $A_\ell(t; \omega_n)$, we can determine the full state of the system, including the surface brightness perturbations.

Our choice to construct the transfer function using a purely real forcing results in a sine and cosine component of the solution for the mode amplitude whose respective amplitudes are
$$\mathcal{A}_{\text{c},n}(\omega, \ell) = \frac{\omega}{(\omega_n - \omega)(\omega_n + \omega)} \left(2\rho_0 \frac{\omega}{k_h} (\mathbf{u}_\ell^\dagger)^* \sqrt{2\pi r^2 \frac{1}{\mathcal{P}'}} \right) \Bigg|_{r = r_{\text{force}}}, \tag{29}$$
$$\mathcal{A}_{\text{s},n}(\omega, \ell) = \frac{-i\omega_n}{(\omega_n - \omega)(\omega_n + \omega)} \left(2\rho_0 \frac{\omega}{k_h} (\mathbf{u}_\ell^\dagger)^* \sqrt{2\pi r^2 \frac{1}{\mathcal{P}'}} \right) \Bigg|_{r = r_{\text{force}}}, \tag{30}$$

[5] In the forcing region where we apply this relationship, from $r = 1$ to $r = 1.2$, the density changes by less than a factor of 2, so this approximation is good.

where n refers to the nth eigenvalue or eigenvector, and $\mathbf{u}_\ell^\dagger$ is the dual basis defined in Eqn. 10 of Lecoanet et al. (2019). The transfer function for the photometric variability at the surface due to a single delta function forcing is

$$T_r(\omega, \ell, r_{\rm force}) = \left[\left(\Re\left[\sum_n \widetilde{\Delta m_\ell}(R_*;\omega_n)\mathcal{A}_{{\rm c},n}\right]\right)^2 + \left(\Re\left[\sum_n \widetilde{\Delta m_\ell}(R_*;\omega_n)\mathcal{A}_{{\rm s},n},\right]\right)^2\right]^{1/2}. \tag{31}$$

The derived link between the mode amplitude and the wave luminosity (the transfer function) is only valid if the response is evaluated for many delta function forcings spread over a range of radii. We perform an analogous operation to that in Eqn. 28 of Lecoanet et al. (2019) and evaluate the mean power of the transfer response of $N_F = 200$ radially evenly spaced delta function forcing terms. In principle, it does not matter which radii we force over so long as those radii are in the radiative zone $r > 1$ and the radial extent over which we force is sufficiently large to ensure that each wave is forced away from their nodes. Indeed, we find similar results if we center the forcing window at $r = 1.1$ or $r = 1.2$ and use forcing windows larger than $\Delta r = 0.05$. In this work, we force in the range $r_{\rm force} = [1.005, 1.2]$ and calculate

$$T(\omega, \ell) = A_{\rm corr}\left[\frac{1}{N_F}\sum_{i=1}^{N_f} T_r^2(\omega, \ell, r_{{\rm force},i})\right]^{1/2}, \tag{32}$$

where $A_{\rm corr}$ is an $\mathcal{O}(1)$ amplitude correction factor, similar to that introduced in Lecoanet et al. (2021), which must be calibrated using simulations. In Section 5.3, we find $A_{\rm corr} = 0.4$ faithfully reproduces the results of wave propagation simulations, and we use that value throughout this work. This indicates that, while our forcing amplitude (Eqn. 27) is designed to match the wave luminosity L_w at the forcing radius, our forcing amplitude is incorrect by a factor of $A_{\rm corr}$. We find the same correction factor $A_{\rm corr} = 0.4$ when comparing wave generation and wave propagation simulations with different resolutions (hence different levels of turbulence). This is likely due to a discrepancy between the boundary and volumetric forcing models used to derive equation 27.

The photometric variability at a given ℓ and ω can then be constructed from the wave luminosity and transfer function,

$$|\Delta m|(\omega, \ell) = T(\omega, \ell)\sqrt{L_w(\omega, \ell)}. \tag{33}$$

5. VERIFICATION OF THE TRANSFER FUNCTION IN WAVE PROPAGATION SIMULATIONS

5.1. *Surface perturbations of standing gravity waves*

We sample the entropy perturbations at the surface of the wave propagation simulation every 30 minutes over the course of the last ~ 1.7 years of simulated time[6]. We plot the amplitude spectrum of those entropy perturbations in Supplementary Fig. 4. In the left panel of Supplementary Fig. 4, we plot the frequency spectrum of the entropy perturbations of the first three spherical harmonics. We see that the shape of the signal at each ℓ is characterized by three features: (1) high-amplitude resonant peaks at high frequency, (2) the imprinted spectrum of convective driving which decreases in amplitude as the frequency increases, and (3) strong radiative damping of gravity waves at low frequencies. In the right panel of Supplementary Fig. 4, we show the quadrature sum of the three signals in the left panel (yellow line) as well as the quadrature sum over all ℓ values in this simulation (black line). When summing over all ℓ values, we observe a relatively flat noise-like signal. We note that the power at high ℓ would not be visible in photometric observations due to cancellation over a single hemisphere, but we still include them in our calculations. In total, this simulation was run for ~ 5 years to allow the power in the standing modes to develop; most of the modes, particularly low-frequency modes, have much shorter lifetimes than the simulation duration and saturate quickly. We choose to run the simulation for so long to ensure that the simulation duration is on the order of the longest mode lifetime, which are the lifetimes of the highest frequency waves (~ 10 years).

5.2. *Calculation of the transfer function for the Wave Propagation simulation*

We now calculate a transfer function which describes how the environment of the Wave Propagation simulation modifies the convective luminosity. To do so, we calculate the eigenvalues and eigenfunctions associated with the gravity waves in the simulation's wave cavity using `Dedalus` and remove spurious numerical eigenvalues using the

[6] Recall from Supplementary Table 2 that the characteristic dynamical timescale of convection is $t_{\rm heat} = 4.66 \times 10^5$ s ≈ 5.4 days.

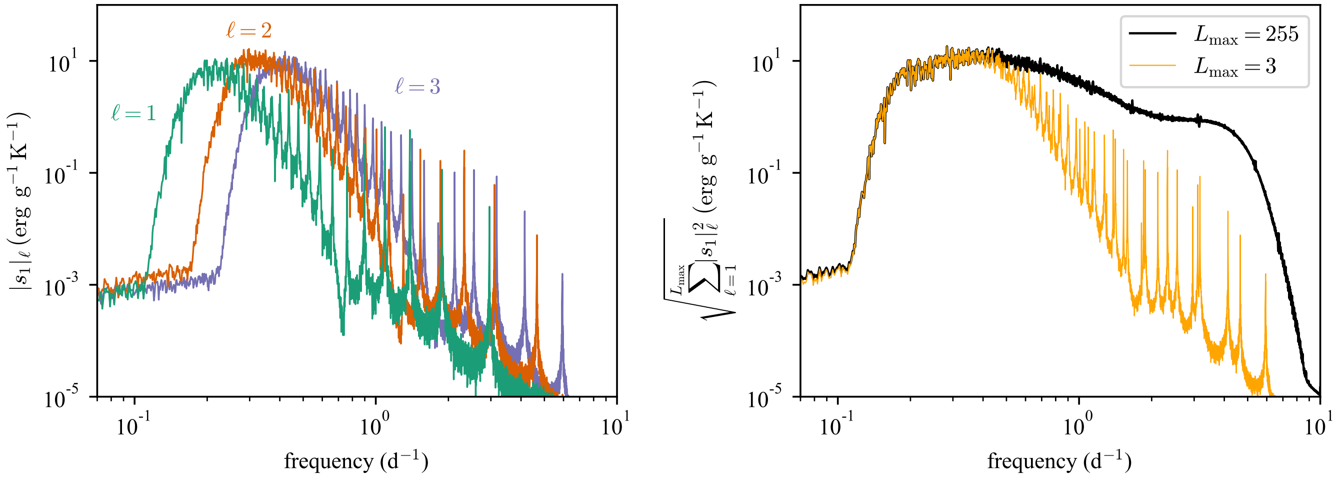

Supplementary Figure 4. The amplitude spectrum of entropy perturbations at the outer boundary of the Wave Propagation simulation. (Left panel) individual contributions to the surface amplitude spectrum from the first three spherical harmonics. (Right panel) Summed entropy amplitude fluctuations over (orange) the first three spherical harmonics and (black) all spherical harmonics. Note that these sums account for all power over the full 4π of the simulation surface; "realistic" observations observing only one hemisphere must account for cancellation effects, see Sec. 6.

approach laid out in Ch. 7 of Boyd (2001) and the `eigentools` package (Oishi et al. 2021). We then calculate the transfer function according to Eqn. 31, but we replace the surface magnitude eigenfunction values $\widetilde{\Delta m_\ell}(R_*, \omega_n)$ with the entropy eigenfunction values at the outer simulation boundary $\widetilde{s_\ell}(R_{\max}, \omega_n)$.

5.2.1. *Timestepping errors in high-frequency waves and Wave Propagation simulation transfer functions*

Before directly computing the transfer function, we first note that timestepping errors in simulations can alter the eigenvalues of gravity waves which propagate in the simulation domain. We must account for these errors before computing the transfer function associated with our simulation.

We evolve the simulations in this paper using `Dedalus`' SBDF2 timestepper, which is the second order semi-implicit backward difference scheme presented in Wang & Ruuth (2008). Timestepping truncation errors can modify a wave's oscillation frequency and damping rate, and this can affect the measured signal of waves in our simulations. A simple damped wave with complex eigenfrequency $\tilde{\omega} = \omega - i\gamma$ evolves according to

$$\partial_t u = -i\tilde{\omega} u \qquad \Rightarrow \qquad \frac{1}{\Delta t}\left(\frac{3}{2}U^n - 2U^{n-1} + \frac{1}{2}U^{n-2}\right) = -i\tilde{\omega} U^n, \tag{34}$$

where U^n is the solution U at time step n.

To approximate the trajectory of the numerical solution, we seek an effective $\tilde{\omega}_{\rm eff}$ such that

$$U^n = \left(e^{-i\tilde{\omega}_{\rm eff}\Delta t}\right)^n U^0 = e^{-i\tilde{\omega}_{\rm eff} n\Delta t} U^0. \tag{35}$$

Substituting this form into Eqn. 34, we obtain a quadratic polynomial in $e^{-i\omega_{\rm eff}\Delta t}$. We take the principal root and Taylor expand the solution in the limit $|i\omega|\Delta t < 1$ to obtain

$$\tilde{\omega}_{\rm eff} = \tilde{\omega} - \frac{1}{3}\tilde{\omega}^3\Delta t^2 - i\frac{1}{4}\tilde{\omega}^4\Delta t^3 + \mathcal{O}(\Delta t^4). \tag{36}$$

By inspecting the magnitude of the real and imaginary parts of Eqn. 36 we can infer information about the effective oscillation frequency and damping rate. When the wave damping timescale is much longer than that of the oscillations, i.e., $0 < \gamma \ll \omega$, we find at leading order that

$$\omega_{\rm eff} = \omega - \frac{1}{3}\omega^3\Delta t^2 + \mathcal{O}(\Delta t^4), \qquad \text{and} \qquad \gamma_{\rm eff} = \gamma + \frac{1}{4}\omega^4\Delta t^3 + \mathcal{O}(\Delta t^5). \tag{37}$$

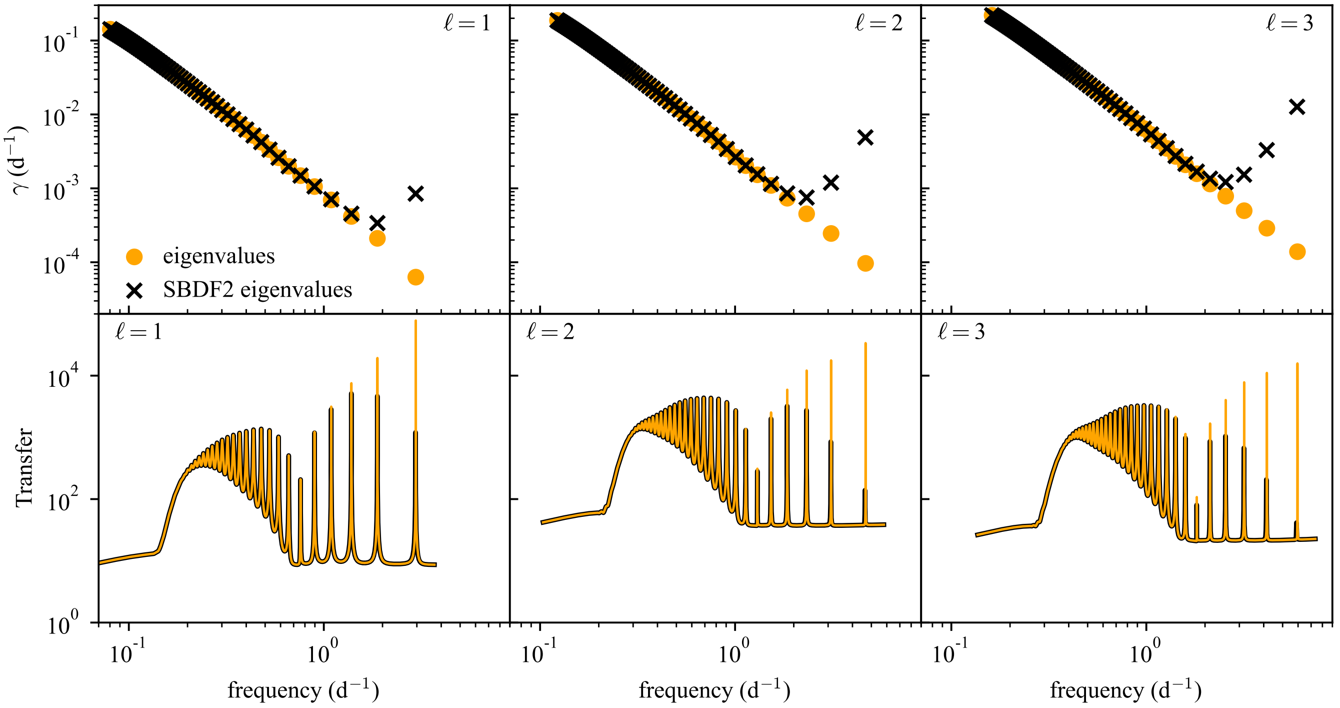

Supplementary Figure 5. The effect of timestepping errors on gravity waves. (Top panels) The spectra of the imaginary vs. real components of the gravity wave eigenvalues for the Wave Propagation simulation for $\ell = 1$ (left), $\ell = 2$ (middle), and $\ell = 3$ (right); orange values are directly obtained from the eigenvalue solve while black values indicate how the orange values change after applying Eqn. 37, assuming $\Delta t = 2.98 \times 10^{-3}$ d, which was a typical timestep size for the Wave Propagation simulation. (Bottom panels) The transfer function calculated using the raw eigenvalues (orange) and the SBDF2 eigenvalues (black).

Thus, in simulations of waves with long damping timescales, Eqn. 37 shows that the effective damping rate $\gamma_{\rm eff}$ is set by ω, which can lead to a drastically enhanced damping rate, sometimes several orders of magnitude larger than the original γ. By contrast, the effective oscillation frequency experiences relatively little change.

In Supplementary Fig. 5, we plot the gravity wave eigenvalues and transfer function associated with the wave cavity in our Wave Propagation simulation for the first three spherical harmonics $\ell = [1, 3]$. In the top panels, we plot the eigenvalues obtained from our eigenvalue solve, and the eigenvalues altered according to Eqn. 36 with $\Delta t = 2.98 \times 10^{-3}$ d, which is a typical timestep size for this simulation. We see that the timestepped solution matches the true eigenvalues for most modes and matches particularly well at low frequency. However, the damping rate of the highest frequency modes is greatly increased. In the case of the $\ell = 1$ modes, we might naively think that the lifetime of the longest-lived wave is $t_{\rm mode} \sim 1/(6 \times 10^{-5})$ d $= 46$ years, but in our timestepped simulation the longest-lived wave actually has a lifetime of $t_{\rm mode} \sim 1/(3 \times 10^{-4})$ d $= 9$ years. For comparison, the duration of our Wave Propagation simulations is ~ 5 years, which allows us to capture a significant portion of the longest mode lifetimes.

The bottom panels of Supplementary Fig. 5 display the transfer functions calculated according to Eqn. 32 using the eigenvalues of the wave cavity (orange lines) and the eigenvalues of the wave cavity accounting for timestepping (black lines); we set $A_{\rm corr} = 0.4$ for this plot, as in the rest of the paper.. We see that the associated changes in the mode lifetimes shown in the top panels also correspond to resonant peaks of much lower quality factor in the transfer function; for example, the quality factor of the peak of the highest frequency wave at $\ell = 3$ decreases by about three orders of magnitude, which is equal in magnitude to the change in the mode lifetime. Note also that at $\ell > 1$, the oscillatory portion of the highest frequency waves are also modestly affected by timestepping errors, which can be seen from the slight horizontal offset between the peaks of the yellow and black lines. Finally, we note that while the transfer function calculation here reproduces all of the expected wave features (resonant peaks and damping at low frequency), at very low frequencies the quality of the transfer function solution degrades. Since our mode expansion is finite rather than infinite, the calculation of the dual basis incurs errors which accumulate in the highest radial order

(lower frequency) gravity modes, and these errors appear as a "noisy tail" to the left side of the transfer function; this feature of the signal is not physical. When using the transfer function to compute surface variability spectra, we manually set the value of the transfer function to be zero at low frequencies where this noisy tell is present, which is a good approximation for these heavily damped waves.

5.3. *Calibrating $A_{\rm corr}$ and comparing Wave Propagation data with transfer function solution*

In Supplementary Fig. 6, we plot the measured surface entropy fluctuations from the Wave Propagation simulation (black lines) as well as the predicted surface entropy perturbations from Eqn. 33. To create this prediction, we multiply the transfer function (black lines in bottom panels of Supplementary Fig. 5) and the square root of the measured wave luminosity from the Wave Generation simulation (the 15 $M_\odot$ orange line in Supplementary Fig. 3), and we find that a scalar amplitude correction factor $A_{\rm corr} = 0.4$ improves the agreement between the transfer function solution and the Wave Propagation measurements, so we use $A_{\rm corr} = 0.4$ in all surface amplitude predictions in this work. We find excellent agreement between the Wave Propagation simulation data and the wave luminosity filtered through the transfer function solution, particularly at moderately low frequencies where the amplitude of the wave response is maximized. The transfer function captures a portion of the damping cutoff at low frequency, the decay of the spectrum baseline with increasing frequency, and the overall character of the standing mode peaks. We note that at higher values of ℓ, the mode peaks and continuum (troughs) are slightly over-predicted, and the amplitudes of the highest frequency modes at each ℓ are under-predicted, perhaps suggesting that our timestepping correction to the eigenmodes overestimates numerical damping in the simulation. However, if we had instead neglected to make the timestepping corrections introduced in Section 5.2.1, the amplitude of the highest-frequency peaks would have been vastly overestimated (e.g., the highest-frequency peaks in orange at $\ell = 1$ and 2 would have amplitudes at least an order of magnitude higher than the peaks in black). Furthermore, standing mode peaks in the wave luminosity

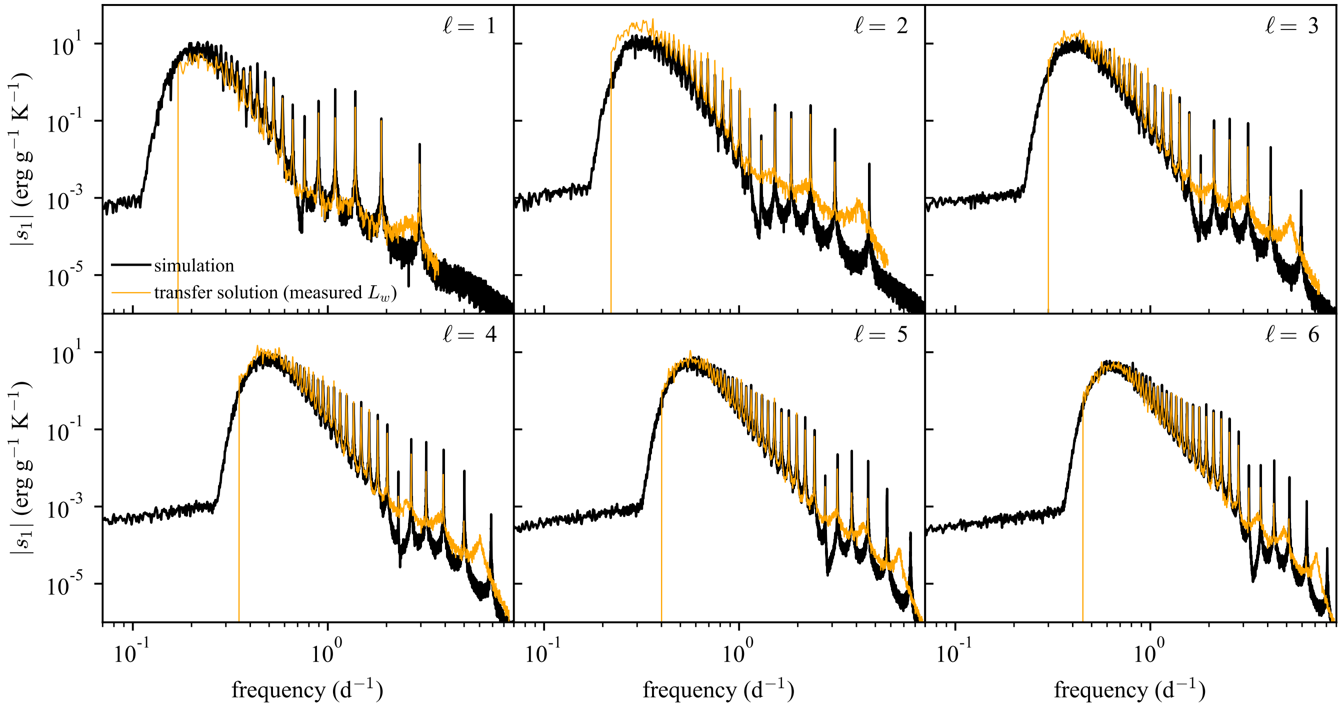

Supplementary Figure 6. Comparison between measured wave signal at simulation surface and transfer function prediction using raw wave luminosity data. Shown are entropy amplitude perturbations at the surface of the Wave Propagation simulation (black lines) for the first six spherical harmonic degrees. Overplotted in orange are our predictions for the surface perturbations. We create the orange line by multiplying the transfer function (shown in bottom panels of Supplementary Fig. 5) by the square root of the wave luminosity from the $15 M_\odot$ Wave Generation simulation shown in Supplementary Fig. 3 and then mulitplying by a uniform $A_{\rm corr} = 0.4$. Note that the transfer functions have unphysical "tails" on their low-frequency edge; we set the transfer function to zero where this tail appears.

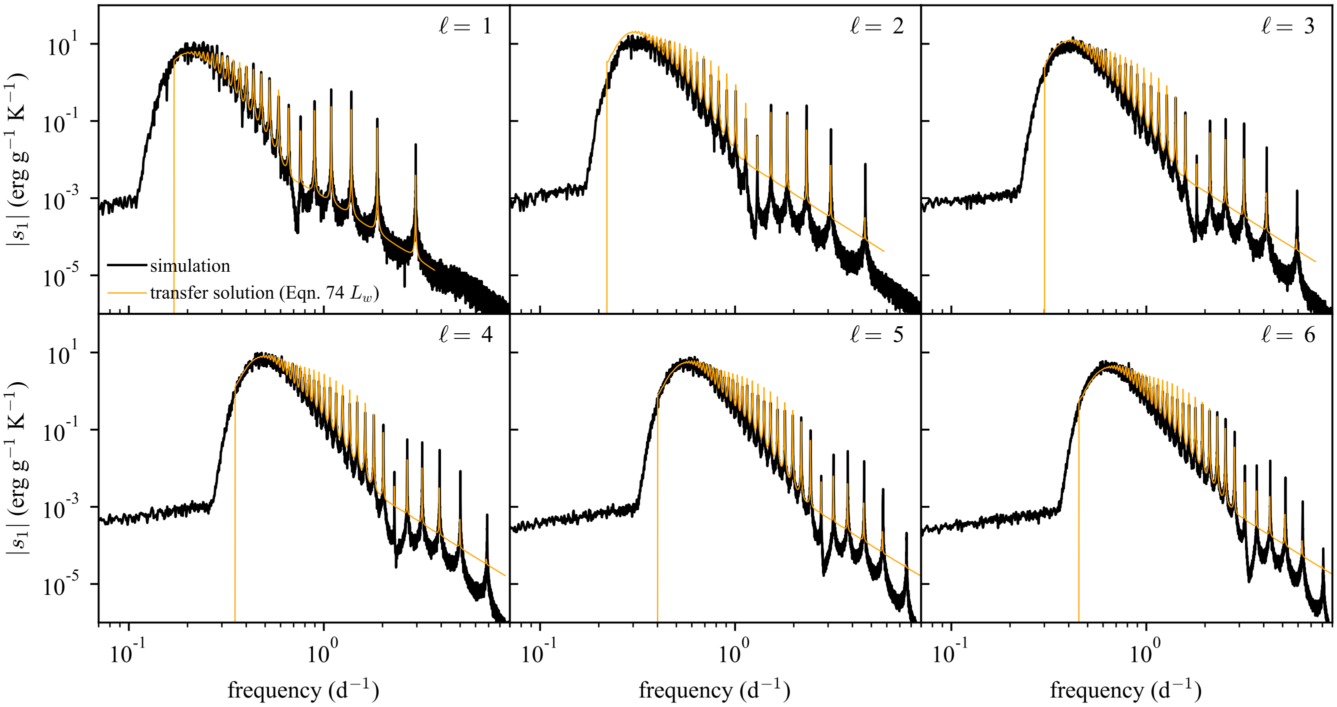

Supplementary Figure 7. Comparison between measured wave signal at simulation surface and transfer function prediction using fitted wave luminosity data. As in Supplementary Fig. 6, but now we create the transfer function prediction by multiplying the transfer function by the best-fit to the wave luminosity shown in Eqn. 20.

spectrum that are not perfectly damped by our damping layers result in unphysical bumps in the predicted solution at high ℓ (see e.g., at $f \gtrsim 0.2$ d^{-1} for $\ell = 4$).

In Supplementary Fig. 7, we plot the same quantities as in Supplementary Fig. 6, but this time we convolve the transfer function with the powerlaw fit to the wave flux from Eqn. 20. Again, we find good agreement between the transfer function and the surface power of our simulations, particularly at $\ell = 1$. We find that as ℓ increases and at higher frequencies, the powerlaw wave luminosity overestimates the wave driving observed in our simulations; perhaps at higher ℓ, the true wave luminosity spectrum is steeper than the $f^{-13/2}$ spectrum we employ. Fortunately, the broad low-frequency "bump", which is the most observable feature in the spectrum, is captured well for all ℓ values.

6. PHOTOMETRIC VARIABILITY SPECTRA

6.1. *The transfer function*

We use `GYRE` and `MSG` to calculate the surface luminosity perturbations associated with each gravity wave eigenmode, then we generate a transfer function according to Eqn. 32 at each ℓ. Transfer functions for $\ell = [1, 2, 3]$ for each of our 3, 15, and 40 $M_\odot$ models studied in this work are shown in Supplementary Fig. 8. In order to retrieve the spectrum of photometric variability at the surface of the star in μmag, we will multiply these transfer functions by the square root of the wave luminosity (Eqn. 20).

For comparison, we also show transfer functions for $10^6 s_1/c_p$ from the Wave Propagation simulation in the middle panels of Supplementary Fig. 8. We see that even though our Wave Propagation simulation captures 93% of the stellar radius and 99.99925% of the stellar mass, the signal at the surface of our simulation is quite different from that at the surface of the star. In particular, the transfer function in our simulations has a low-frequency damping tail which occurs at too high of a frequency compared to the full star, so low frequency waves are damped too heavily in our simulations compared to the star. Our simulation transfer functions do have high-frequency peaks at frequencies which are very close to the oscillation frequencies present in the real star, which makes sense as the bulk of the high-frequency wave cavity is deep in the star and these waves are evanescent near the surface. However, the quality factor of the wave peaks in our simulated transfer functions are much lower than they are for the full star due to the simulation diffusivities and excess damping from timestepping; we do not apply the timestepping correction laid out Sec. 5.2.1

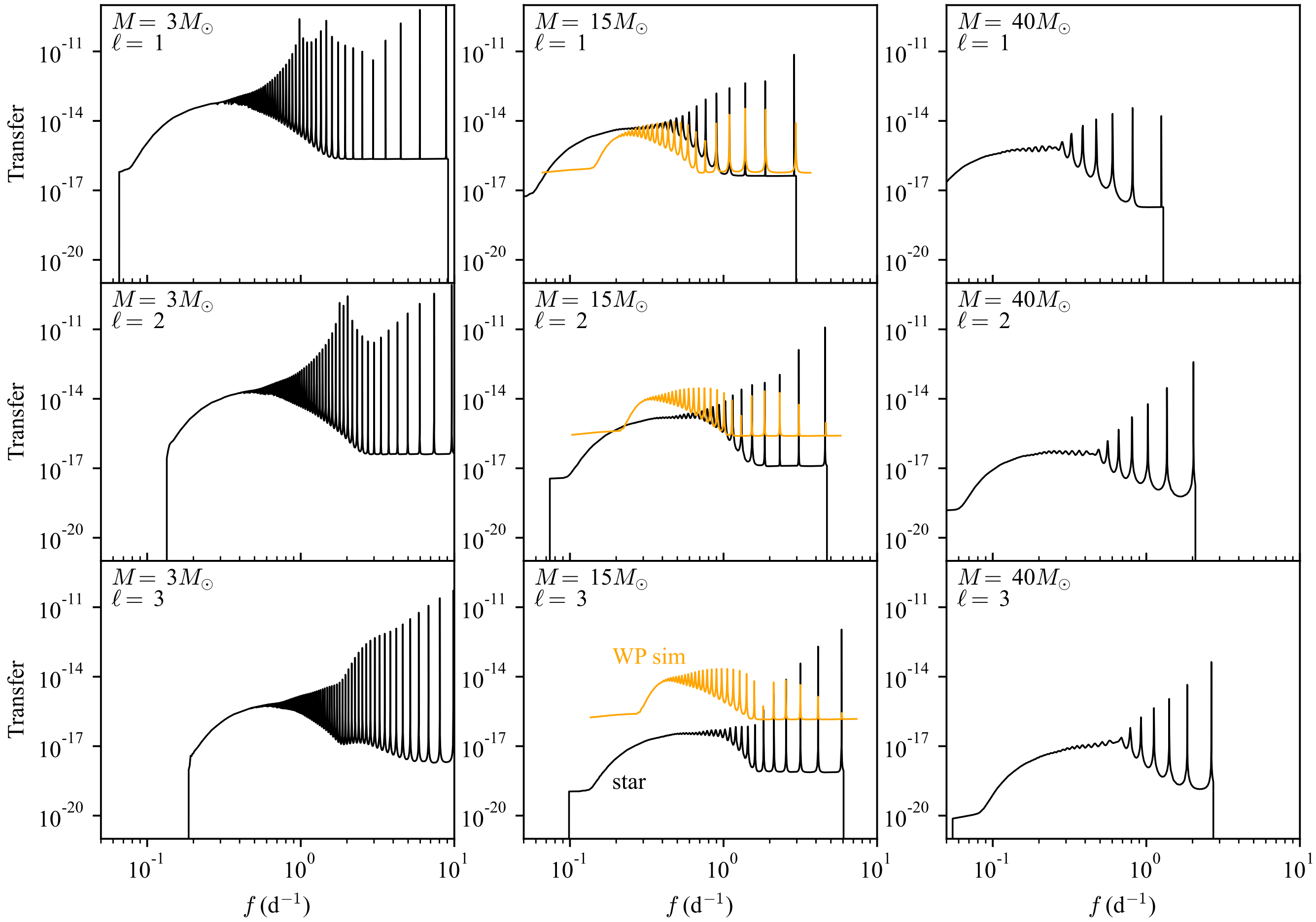

Supplementary Figure 8. The frequency spectra of the transfer function in each of the stars studied. We plot the transfer functions for (top row) $\ell = 1$, (middle row) $\ell = 2$, and (bottom row) $\ell = 3$ for our (left column) $3M_\odot$, (middle column) $15M_\odot$, and (right column) 40 $M_\odot$ stars. Black lines show the transfer function for converting the square root of the gravity wave luminosity into photometric variability in μmag at the stellar photosphere. The orange lines in the middle columns show comparable transfer functions for the Wave Propagation simulation. As ℓ increases, the stellar transfer functions fall in amplitude compared to the simulation transfer functions; this occurs because the stellar transfer functions include wave cancellation and limb darkening effects which are incurred when observing a hemisphere for comparison with observations, while the simulation transfer functions do not.

to the GYRE eigenmodes when creating the transfer functions used in our predictions of the observable photometric variability.

Note that the transfer function for the star decreases sharply in magnitude as ℓ increases while the simulation transfer function does not. This occurs because when we use MSG to calculate $\widetilde{\Delta m}_{\ell,n}$, we account for the effects of limb darkening and cancellation; these effects result in smaller photometric variability perturbations from modes at high spherical harmonic degree. We do not include these in our simulations, where the transfer function gives the amplitude expected for the wave over the full 4π.

6.2. *Synthesis of photometric variability spectra*

We generate a transfer function according to Eqn. 32 and use it to filter the wave luminosity to predict the surface photometric variability for each spherical harmonic $\ell = [1, 15]$ according to Eqn. 33 and using the wave luminosity fits in Eqn. 20. We sample the synthesized variability in uniformly spaced frequency bins $\Delta f = 3.17 \times 10^{-8}$ Hz up to $f_{\rm max} = 5.55 \times 10^{-4}$ Hz, corresponding to a year of observational data sampled every 30 minutes. We plot the magnitude contributions of each ℓ for each star in the left panels of Supplementary Fig. 9. We plot partial quadrature sums of the total observed photometric variability for each star in the right panels of Supplementary Fig. 9. The contributions

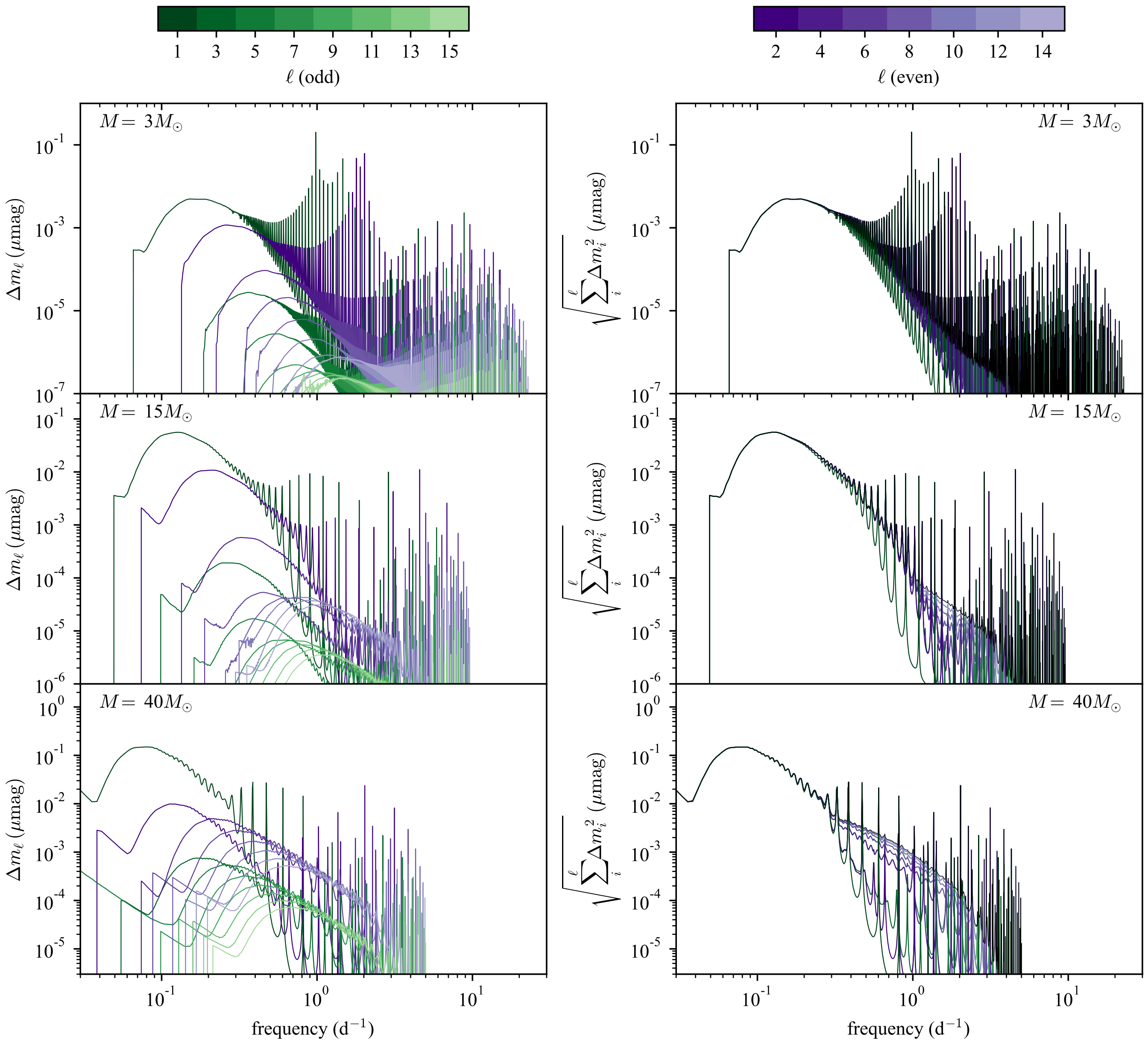

Supplementary Figure 9. Contributions to photometric variability from waves of each spherical harmonic degree. (Left panels) Shown are the photometric variability magnitude contributions for each spherical harmonic degree for the 3 $M_\odot$ (top panel), 15 $M_\odot$ (middle panel), and 40 $M_\odot$ (bottom panel) stars. Odd ℓ lines are colored green and decrease in saturation as ℓ increases; even ℓ lines are similar but are colored purple. In the right panels, we correspondingly show partial quadrature sums of the full photometric variability signal from $\ell = 1$ to the ℓ value corresponding to the line color. The black lines are the sums over all plotted ℓ values and are the same lines as those plotted in the middle panel of Fig. 2 in the main text.

of odd spherical harmonic degrees are shown as green lines, and the contributions of even spherical harmonic degrees are shown as purple lines. We find that the dominant contributions are from the $\ell = 1$ mode, and aside from that the even modes contribute more powerfully to the observed spectra than the odd modes, which experience more cancellation. In most transfer functions, we see a "tail" at low frequency where the the transfer function increases with decreasing frequency. This "tail" is a numerical artifact which arises because the eigenfunction expansion breaks down for low frequencies for gravity waves of high radial order, making the transfer function calculation unreliable for low frequencies. We know that the transfer function should be falling off exponentially in this regime due to radiative diffusivity, so it is not a bad approximation to set it equal to zero here, which we do.

From the right-hand panels, we see that the majority of the observed signal is caused by the $\ell = 1$ contribution. At higher frequencies, the contributions of higher ℓ values increase the magnitude of the "tail" of the gravity wave signal. The main broad peak of the signal is therefore saturated by our inclusion of $\ell = [1, 15]$, but a small amount of power would continue to be added at high frequencies if we were to include all spherical harmonic degrees. We find that for $\ell > 15$, `GYRE` has trouble faithfully reconstructing the eigenfunctions, and so we are limited to the values of ℓ studied here.

We briefly note that our predictions and theory are predicated upon the gravity waves remaining linear. While the waves remain linear in our Wave Propagation simulation, those simulation domains only extend up to 93% of the stellar radius. The transfer function Eqns. 29 & 30, when multiplied by $\sqrt{L_w}$, gives any perturbation variable at any radial location. We use these equations to determine the amplitude of the horizontal velocity eigenfunctions $u_{h,n}$ of each eigenvalue n within the range of radial coordinates that are not included in the Wave Propagation simulation, $r \in [0.93, 1]R_*$. We calculate the nonlinearity per Eqn. 14 of Ratnasingam et al. (2019),

$$\epsilon_n = \frac{u_{h,n}}{\omega_n} k_h, \tag{38}$$

where ω_n is the real angular frequency of the wave and $k_h = \sqrt{\ell(\ell+1)}/r$ is the horizontal wavenumber where we measure the nonlinearity. We find that $\epsilon_n < 2 \times 10^{-3}$ for all $\ell = 1$ eigenmodes in the 15 $M_\odot$ fiducial star. Since $\epsilon_n \ll 1$, our predicted wave amplitudes correspond to linear waves in the surface layer of the stars, and applying our transfer function theory to predict the magnitude at the stellar surface is consistent. Our finding that the waves are linear near the surface of the star is consistent with the recent results presented in Section 5 of Le Saux et al. (2023), who find that the wave nonlinearity is greatest near the convection zone, not the stellar surface, because waves are strongly radiatively damped near the surface.

6.3. *Red noise fits to synthesized spectra*

In Supplementary Fig. 10, we show Lorentzian fits (orange lines) of the form,

$$\alpha(\nu) = \frac{\alpha_0}{1 + \left(\frac{\nu}{\nu_{\text{char}}}\right)^\gamma}. \tag{39}$$

to our photometric magnitude predictions (black lines). Fits are made by-eye with the goal of having the fit describe the peak amplitude of the wave signal and to have the Lorentzian tail follow the tail of the photometric variability spectrum, ignoring the resonant peaks.

7. THE EFFECTS OF ROTATION

7.1. *Observations of stellar rotation and red noise*

In the left panels of Supplementary Fig. 11, we plot the amplitude of red noise versus $v \sin i$ of observed stars using data from table A2 of Bowman et al. (2020). Data points are colored by T_{eff} and the size of the points correspond to the spectroscopic luminosity $\mathscr{L}$. In the top panels, we plot all points from the Bowman et al. (2020) dataset, but we have discarded stars whose effective temperature and luminosity lie outside of the main sequence according to MESA stellar models from Jermyn et al. (2022a); this discards a few stars just to the left of the model ZAMS and all post-main sequence stars. The points with black outlines are the points with similar T_{eff} and $\mathscr{L}$ to our fiducial 15 $M_\odot$ star, and appear in Fig. 2 of the main manuscript. In the bottom panels of Supplementary Fig. 11, we show zoomed views that focus just on the stars most similar to our fiducial star. We also include an estimate of the photometric variability from one rotating simulation, discussed in Section 7.2 and marked as a star.

The importance of rotation on convective motions depends on both the rotation rate of the star and the characteristic overturn timescale of the convective motions. This is quantified by the Rossby number[7] (e.g., Aurnou et al. 2020),

$$\text{Ro} = \frac{U}{2\Omega L} = \frac{P_{\text{rot}}}{t_{\text{conv}}}, \tag{40}$$

where U is a characteristic convective velocity, L is a characteristic length scale, Ω is the stellar angular rotation frequency, and P_{rot} and t_{conv} are respectively the rotational period and convective timescales. For each star in the

[7] We note that this value of Ro may not describe the aspect ratio of convective features well when Ro < 1 (Vasil et al. 2021).

sample, we calculate a projected rotation period, $P_{\rm rot,p} = 2\pi R_*/(v \sin i)$. We interpolate the convective turnover time of the core convection zone from the MESA models of Jermyn et al. (2022a) in $\mathscr{L}$-$T_{\rm eff}$ space and we determine $t_{\rm conv}$ for each star from the $\mathscr{L}$ and $T_{\rm eff}$. We calculate an approximate projected Rossby number for each star,

$$\mathrm{Ro_p} = \frac{\mathrm{Ro}}{\sin i} = \frac{P_{\rm rot,p}}{t_{\rm conv}}. \tag{41}$$

In the right panels of Supplementary Fig. 11, we plot the magnitude of red noise vs. $\mathrm{Ro_p}$. Here we assume rigid rotation when calculating $\mathrm{Ro_p}$. While quasi-rigid rotation is expected on theoretical grounds (e.g., Heger et al. 2005; Brott et al. 2011), asteroseismic studies of massive stars do show the presence of a moderate degree of radial differential rotation in some targets (Bowman 2020).

We find no clear correlation between projected rotational velocity and red noise amplitude. Even accounting for the convective overturn timescale via the Rossby number, there is no clear trend. We do note that the highest magnitude red noise fluctuations seem to occur on stars with moderately high Rossby number or moderately low $v \sin i$. If rotation were boosting convective wave excitation which were in turn causing red noise, we would expect to see a clear increase of α with increasing $v \sin i$, or likewise a clear decrease in α with increasing $\mathrm{Ro_p}$.

It is important to note that $v \sin i$ contains a degeneracy between the stellar rotational velocity and the star's inclination angle. Augustson et al. (2020) theorize that rotation can boost the wave luminosity significantly, and we expect $v \sin i$ to be a robust indication of whether rotationally-boosted waves could be viewed on a rotating star. If the star is a slow rotator, then $v \sin i$ will be small and our non-rotating theory here should apply. If the star is a rapid rotator, its waves will be confined to the equator. If the waves are equatorially trapped, viewing the star from a polar angle (small $\sin i$) will lead to lower wave photometric variability than viewing the star from an equatorial angle ($\sin i \sim 1$).

7.2. *A $15 M_\odot$ simulation with $P_{\rm rot} = 10$ d*

All stars rotate, and massive stars in particular tend to rotate rapidly (Aerts et al. 2019), so it is important to understand how rotation affects our predictions for the photometric variability of massive stars. There are at least two basic ways that rotation can affect gravity waves generated by core convection: by affecting the character of the convection in the core, or by affecting the waves themselves. Rotation affects convection in the core when the Rossby number $\mathrm{Ro} \sim 1$ or smaller. In rotating convection, convective structures align with the rotation axis forming columns and gaining significant anisotropy and organization (c.f., Aurnou et al. 2020; Featherstone & Hindman 2016). This can

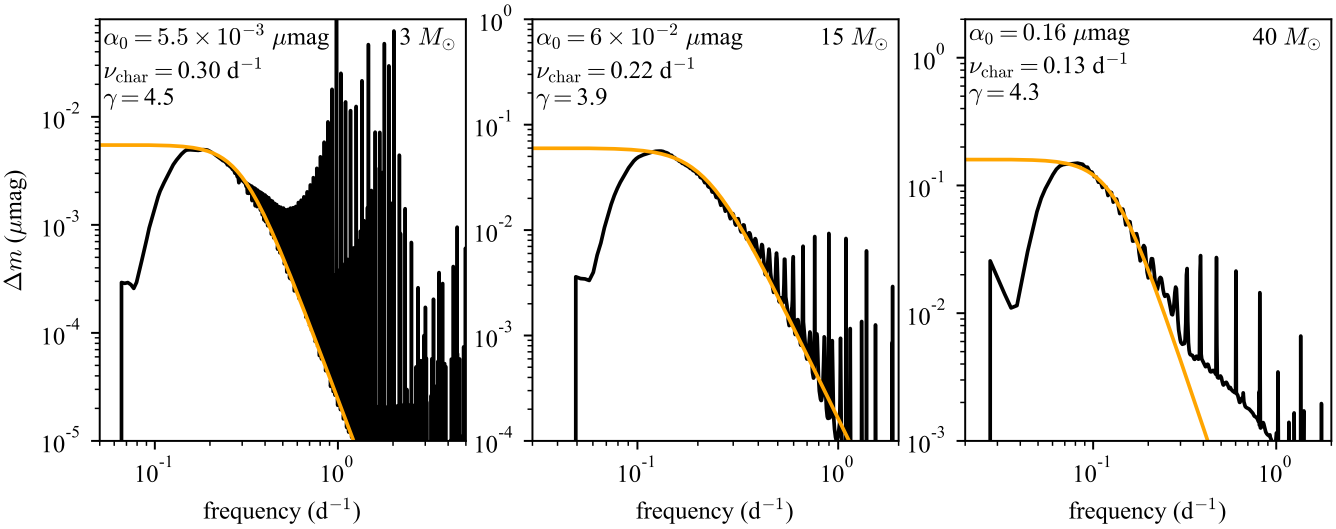

Supplementary Figure 10. Lorentzian fits to predicted photometric variability spectra. We plot the predicted photometric variability spectra of the 3 $M_\odot$ (left), 15 $M_\odot$ (middle), and 40 $M_\odot$ (right) as black lines. We fit a Lorentzian profile as in Eqn. 39 by-eye to the turnover of these spectra at high frequency and overplot these as orange lines. The values used in the Lorentzian fit are reported in the upper left of each panel.

happen even when the rotation frequency is much lower than the wave frequencies, so convection can change character despite the waves being nearly the same as in the non-rotating case. A stably stratified region under the influence of rotation exhibits both gravito-inertial waves and inertial waves (Mathis et al. 2014), so rotation strongly affects the frequencies and structure of oscillation modes with frequencies $\lesssim 2P_{\rm rot}^{-1}$. Properly accounting for rotation in eigenvalue solves and the construction of the transfer function is a difficult task which is beyond the scope of this work.

However, most of the features of the transfer functions for our fiducial 15 $M_\odot$ star occur at frequencies greater than 0.2 d^{-1}, so for rotation periods $P_{\rm rot} \geq 10$ d, using the transfer functions built based on non-rotating modes is a decent first approximation. From the bottom panels of Supplementary Fig. 11, we see that stars similar to our fiducial star have $v \sin i \sim 5 - 30$ km s^{-1}, and these similar stars have a median $v \sin i = 24$ km s^{-1}. Our fiducial 15 $M_\odot$ has $R_* = 4.289\ R_\odot$; therefore a 10-day rotation period produces a surface equatorial rotation velocity of $v = 21.7$ km s^{-1}, which is similar to the median $v \sin i$ in the limited sample of observed stars shown in Fig. 2 of the main manuscript. We therefore run one Wave Generation simulation of our fiducial model with a rotation period of 10 days. The goal of this simulation is not to study wave generation in a rapidly-rotating star, but rather to determine if a modest rotation rate drastically changes the convective generation of waves.

We calculate the convective timescale of our fiducial model according to Eqn. 20 of Jermyn et al. (2022a),

$$t_{\rm conv} = \int_{\rm CZ} \frac{dr}{v_{\rm c}}, \tag{42}$$

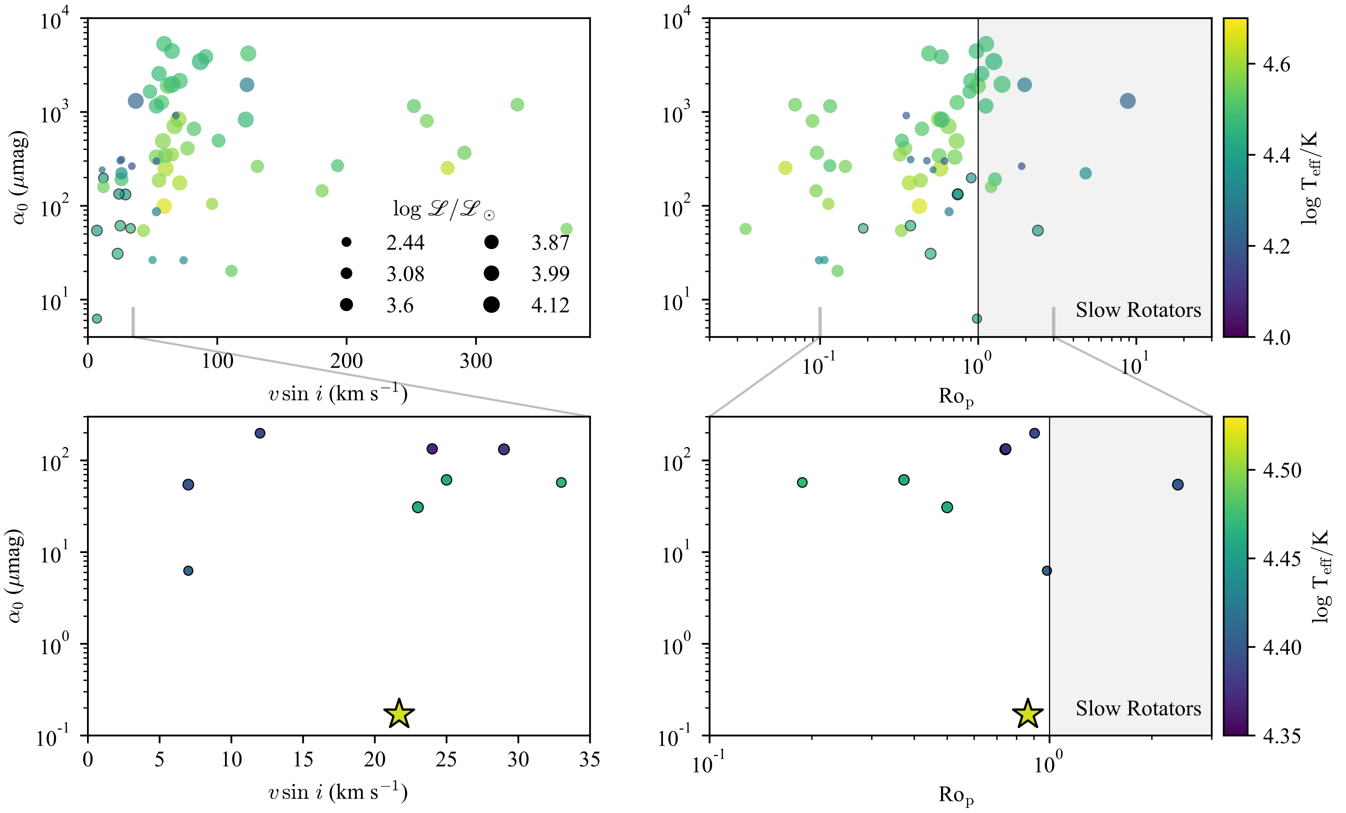

Supplementary Figure 11. No clear correlation between red noise amplitude and stellar rotation rate. (Left panels) Red noise amplitude α_0 vs. projected rotational velocity $v \sin i$ (km / s). Data are colored by $T_{\rm eff}$ and the size of the markers corresponds to the spectroscopic luminosity $\mathscr{L}$ of the star; data are retrieved from tables A1 and A2 of Bowman et al. (2020). (Right panels) Red noise amplitude α_0 vs projected Rossby number Ro$/\sin i$; marker color and size are determined in the same manner as in the left panel. When stars are at or above Ro ~ 1, rotation can affect convection but is not rapid enough to affect the waves themselves substantially. We term these stars "Slow Rotators". The top panels show the full dataset; stars most similar in the $\mathscr{L} - T_{\rm eff}$ plane to our fiducial star are outlined with black and are shown in the bottom panels. The bottom panels additionally contain a star point corresponding to the rotating simulation discussed in Section 7.2; note in the right panels that two stars lie on top of each other near $\mathrm{Ro_p} \approx 0.8$ and $\alpha_0 \approx 200$ μmag.

where v_c is the MLT convective velocity, and find $t_{\rm conv} = 14.75$ d. Taking the average value $|\sin i| = \pi/4$, the approximate projected Rossby number of this simulation is $\mathrm{Ro_p} = \mathrm{P_{rot}}/(\mathrm{t_{conv}}|\sin \mathrm{i}|) = 0.91$. In the rotating simulation, the realized average velocity in the core convection zone is 7.94×10^4 cm s^{-1} (see Supplementary Table 3); this velocity value is larger than in the non-rotating case because a differential rotation that is strong relative to the convective perturbations is established in the azimuthal velocity. The velocity fluctuations around the differential rotation are of order 5.5×10^4 cm s^{-1}, commensurate with the nonrotating velocities and closely matching the MLT estimate for the velocity of 6.68 $\times 10^4$ cm s^{-1} given in Supplementary Table 2; therefore estimating this simulation to have $\mathrm{Ro_p} \approx 1$ is a reasonable approximation. The convective flows in this simulation are therefore influenced by rotation, but are not strongly constrained by it.

We display volume visualizations of the radial velocity of the rotating simulation and the comparison nonrotating simulation in Supplementary Fig. 12. Note that in the nonrotating simulation (bottom panels), the convective flows predominantly form a large dipole flow; this flow migrates around the simulation over time, and the regions of most intense wave generation occur near the impacting site of the dipole and shear regions of the return flow (Herwig et al. 2023). In the rotating simulation, there is no dominant dipole flow; rather, the convective structures are elongated along the rotation axis and swept azimuthally by a strong differential rotation. In the rotating simulations, the wave generation seems to primarily be located near the pole. We refer the reader to the animated version of Supplementary Fig. 12, where these phenomena are more clear than in a static image.

We now compare the wave luminosity of the rotating and nonrotating simulations in the top four panels of Supplementary Fig. 13. We examine the wave luminosities for $\ell = \{1, 2\}$ and for $f = \{0.4, 0.8\}$ d^{-1}. Despite the different flow morphologies displayed in Supplementary Fig. 12, we find that the wave luminosity spectra in the rotating simulation can still be described by a $f^{-6.5}k_h^4$ powerlaw (Lecoanet & Quataert 2013), but the rotating simulation has a slightly higher wave luminosity. We approximate the wave luminosity spectrum in the rotating simulation as

$$L_w = (3 \times 10^{-10}\,\mathrm{erg\,s^{-1}}) \left(\frac{f}{\mathrm{Hz}}\right)^{-6.5} k_h^4, \tag{43}$$

with $k_h = \sqrt{\ell(\ell+1)}$; this fit is shown by the black line in the top four panels of Supplementary Fig. 13 and is a factor of 13 larger than the nonrotating wave flux displayed in Eqn. 20.

We next approximate the surface variability of the rotating simulation. For convenience, we use the nonrotating transfer functions computed in Sec. 6; this might be a decent approximation if the stellar eigenmodes are only weakly modified by rotation . Although we do not study it here, rotation influences the transfer function in two ways. First, the Coriolis force causes m-dependent splitting of wave modes (Aerts et al. 2010, Eqn. 3.360). Second, shifting from the rotating frame of reference to the inertial frame of the observer also causes m-dependent splitting (Aerts et al. 2010, Eqn. 3.315). Including splitting would likely broaden the transfer function, but it would also serve to further decrease the amplitude of the signal, because the power from the different m contributions is spread out rather than concentrated at a single frequency. The gravity waves which are most strongly affected by rotation have $f < 2P_{\rm rot}^{-1}$; we do not observe many waves at $\ell \geq 2$ at those low frequencies in our rotating simulation (Supplementary Fig. 13). We produce a photometric variability spectrum using the nonrotating transfer function (Supplementary Fig. 8, middle panels) and the wave luminosity fit from the rotating simulation (Eqn. 43), and we compare this spectrum and the nonrotating spectrum in the bottom left panel of Supplementary Fig. 13. As expected, the increase in the wave luminosity by about an order of magnitude shifts the photometric variability upwards by about half an order of magnitude, but the surface magnitude fluctuations are still very small, less than $0.5\,\mu$mag. In the bottom right panel of Supplementary Fig. 13, we plot a Lorentzian fit to this spectrum; this is the fit used to determine the α_0-value of the rotating simulation in the bottom panels of Supplementary Fig. 11.

In summary, we find that a moderate rotation is able to modestly boost the wave luminosity, but not to the point where the photometric variability is observable. Critically, we find that this conclusion holds even when the core convection itself is substantially affected, changing from dipole dominated in the non-rotating case to columnar convection in the Ro ~ 1 case. This gives us substantial confidence in our overall findings, that convectively driven waves are not the source of red noise photometric variability, for moderately rotating stars.

Many stars rotate more rapidly than the star that we simulate, and since rotation can boost the wave luminosity it is plausible that photometric variability due to convectively-driven gravity waves may reach observable magnitudes in very rapidly rotating stars. Future work should determine how to properly build transfer functions from rotating eigenvalues and eigenvectors, and should also understand how the wave luminosity varies with decreasing Ro.

While red noise is ubiquitous (Bowman et al. 2019), not all stars are rapid rotators. The low amplitude that we predict in our modestly rotating and nonrotating simulations rule out gravity waves driven by core convection as the source of this ubiquitous signal which is present both in very slow and very rapid rotators (Supplementary Fig. 11,

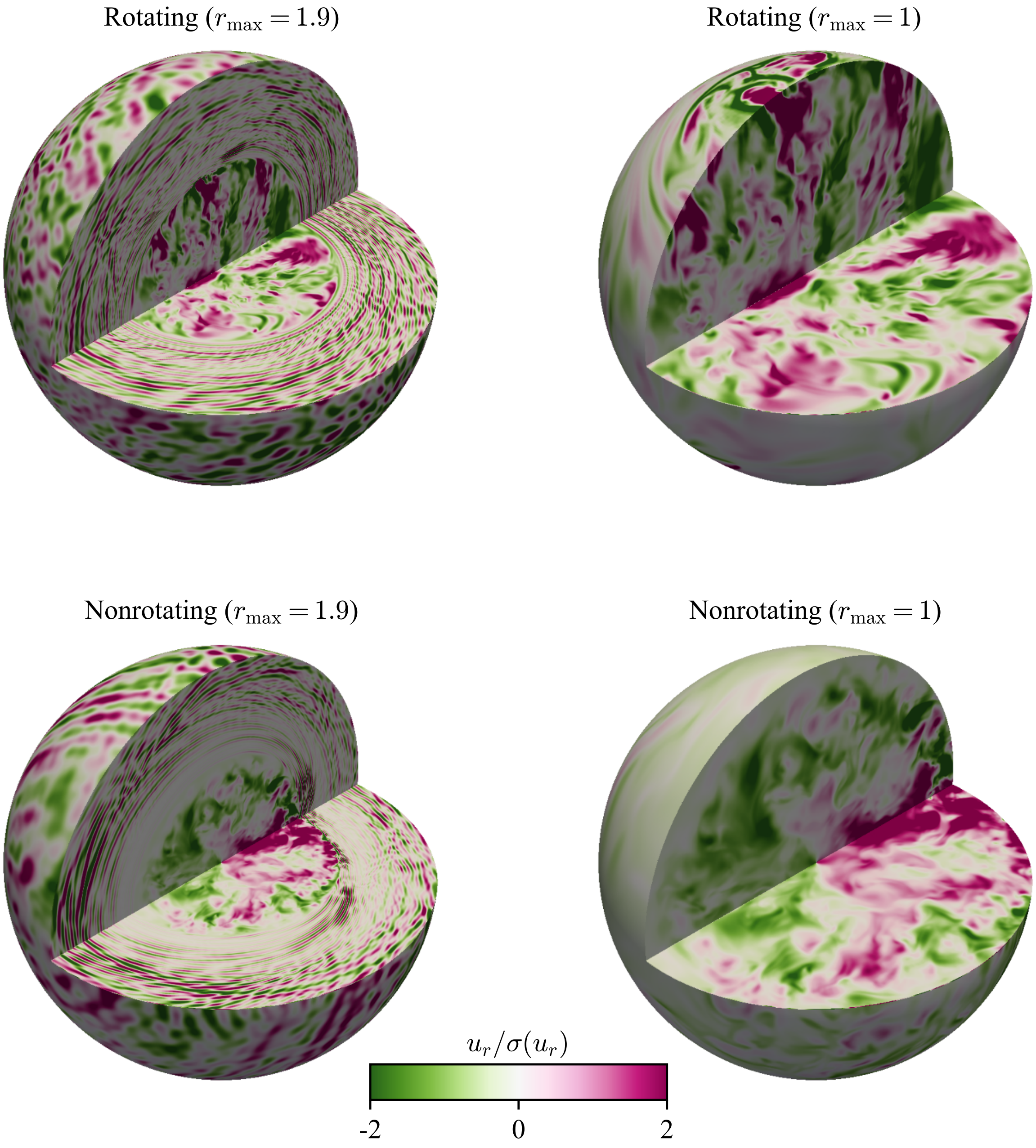

Supplementary Figure 12. Visualizations of flows in both rotating and nonrotating simulations. (Top panels) Volume visualizations of the rotating simulation including (left panel) 95% of the simulation domain and (right panel) 50% of the simulation domain; the right panel focuses on the convection zone while the left panel shows the dynamics throughout the simulation. Bottom panels are as in the top panels, but for the nonrotating case. As in the middle panel of Fig. 1 of the main manuscript, the plotted field is the radial velocity normalized by the standard deviation of the radial velocity at each radius. This figure was created using `PyVista` version 0.37.0 (Sullivan & Kaszynski 2019). An animated version of this still image can be found online in supplemental movie `15msol_rotating_and_nonrotating.mp4`.

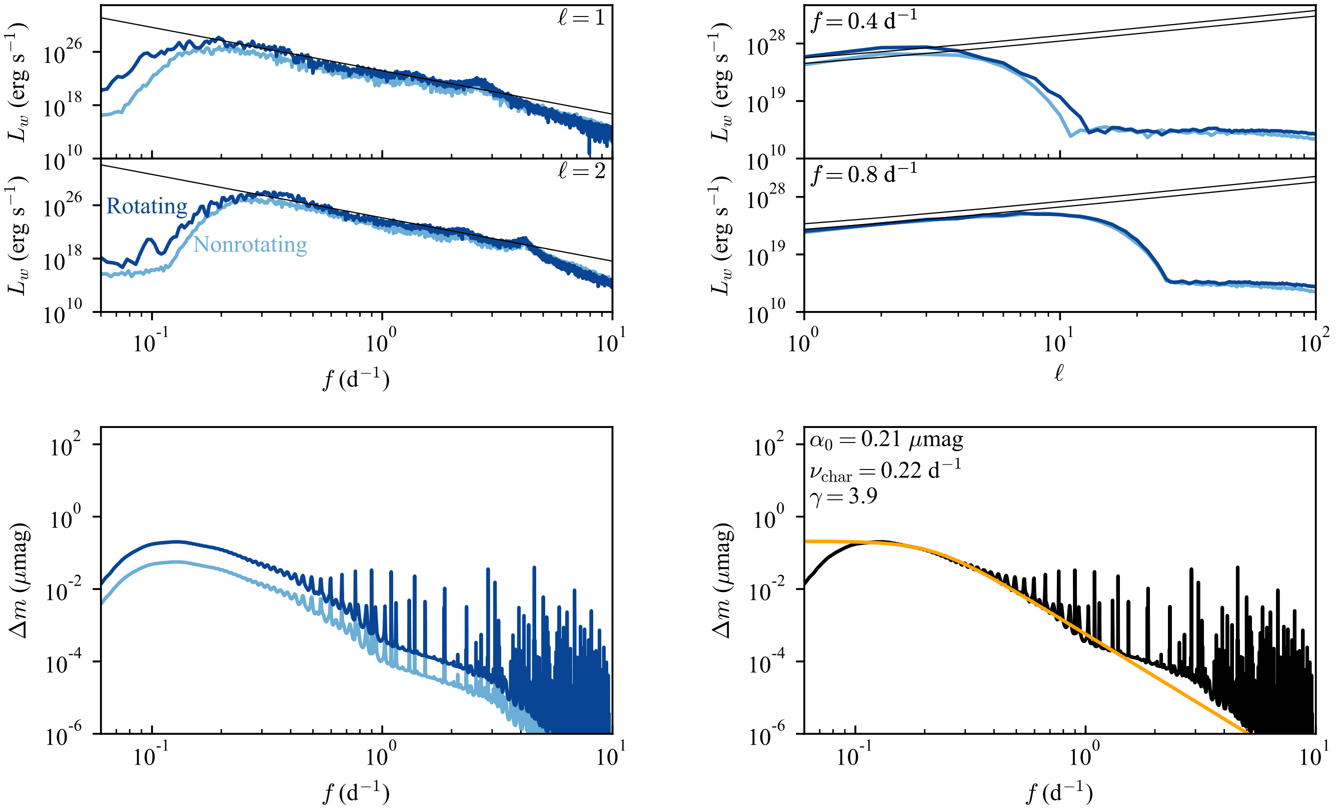

Supplementary Figure 13. Wave luminosity and predicted photometric variability from the rotating simulation. (Top four panels) The wave luminosity as in Supplementary Fig. 3, but here we compare the wave luminosity of the rotating and nonrotating simulations of the 15 $M_\odot$ star. The black line shows the fit from Eqn. 43. (Bottom left panel) Summed photometric variability spectra, as shown in Fig. 2 of the main text, here for the rotating and nonrotating 15 $M_\odot$ simulations. (Bottom right panel) Red noise fit, as in Supplementary Fig. 10, but here for the rotationally-boosted variability spectrum shown in the bottom left panel.

upper right panel). On the other hand, the observed insensitivity of red noise amplitude with stellar rotational velocity is expected if subsurface convection is the source of red noise, since the near-surface convective regions in OB stars are always in the regime of Ro > 1 (Cantiello et al. 2021; Jermyn et al. 2022a).

REFERENCES

Aerts, C., Christensen-Dalsgaard, J., & Kurtz, D. W. 2010, Asteroseismology, doi: 10.1007/978-1-4020-5803-5

Aerts, C., Mathis, S., & Rogers, T. M. 2019, ARA&A, 57, 35, doi: 10.1146/annurev-astro-091918-104359

Anders, E. H., & Brown, B. P. 2017, Physical Review Fluids, 2, 083501, doi: 10.1103/PhysRevFluids.2.083501

Anders, E. H., Lecoanet, D., Cantiello, M., et al. 2023, Code, Data, and Supplemental Materials for "The photometric variability of massive stars due to gravity waves excited by core convection", 1.1, Zenodo, doi: 10.5281/zenodo.7764997

Angulo, C., Arnould, M., Rayet, M., et al. 1999, NuPhA, 656, 3, doi: 10.1016/S0375-9474(99)00030-5

Augustson, K. C., Mathis, S., & Astoul, A. 2020, ApJ, 903, 90, doi: 10.3847/1538-4357/abba1c

Aurnou, J. M., Horn, S., & Julien, K. 2020, Physical Review Research, 2, 043115, doi: 10.1103/PhysRevResearch.2.043115

Blouin, S., Shaffer, N. R., Saumon, D., & Starrett, C. E. 2020, ApJ, 899, 46, doi: 10.3847/1538-4357/ab9e75

Bowman, D. M. 2020, Frontiers in Astronomy and Space Sciences, 7, doi: 10.3389/fspas.2020.578584

Bowman, D. M., Burssens, S., Simón-Díaz, S., et al. 2020, A&A, 640, A36, doi: 10.1051/0004-6361/202038224

Bowman, D. M., Burssens, S., Pedersen, M. G., et al. 2019, Nature Astronomy, 3, 760, doi: 10.1038/s41550-019-0768-1

Boyd, J. P. 2001, Chebyshev and Fourier Spectral Methods

Brott, I., de Mink, S. E., Cantiello, M., et al. 2011, A&A, 530, A115, doi: 10.1051/0004-6361/201016113

Burns, K. J., Vasil, G. M., Oishi, J. S., Lecoanet, D., & Brown, B. P. 2020, Physical Review Research, 2, 023068, doi: 10.1103/PhysRevResearch.2.023068

Cantiello, M., Lecoanet, D., Jermyn, A. S., & Grassitelli, L. 2021, ApJ, 915, 112, doi: 10.3847/1538-4357/ac03b0

Cassisi, S., Potekhin, A. Y., Pietrinferni, A., Catelan, M., & Salaris, M. 2007, ApJ, 661, 1094, doi: 10.1086/516819

Chugunov, A. I., Dewitt, H. E., & Yakovlev, D. G. 2007, PhRvD, 76, 025028, doi: 10.1103/PhysRevD.76.025028

Couston, L.-A., Lecoanet, D., Favier, B., & Le Bars, M. 2018, Journal of Fluid Mechanics, 854, R3, doi: 10.1017/jfm.2018.669

Currie, L. K., & Browning, M. K. 2017, ApJL, 845, L17, doi: 10.3847/2041-8213/aa8301

Cyburt, R. H., Amthor, A. M., Ferguson, R., et al. 2010, ApJS, 189, 240, doi: 10.1088/0067-0049/189/1/240

Featherstone, N. A., & Hindman, B. W. 2016, ApJL, 830, L15, doi: 10.3847/2041-8205/830/1/L15

Ferguson, J. W., Alexander, D. R., Allard, F., et al. 2005, ApJ, 623, 585, doi: 10.1086/428642

Fuller, G. M., Fowler, W. A., & Newman, M. J. 1985, ApJ, 293, 1, doi: 10.1086/163208

Goldreich, P., & Kumar, P. 1990, ApJ, 363, 694, doi: 10.1086/169376

Heger, A., Woosley, S. E., & Spruit, H. C. 2005, ApJ, 626, 350, doi: 10.1086/429868

Herwig, F., Woodward, P. R., Mao, H., et al. 2023, arXiv e-prints, arXiv:2303.05495, doi: 10.48550/arXiv.2303.05495

Howell, S. B., Sobeck, C., Haas, M., et al. 2014, PASP, 126, 398, doi: 10.1086/676406

Iglesias, C. A., & Rogers, F. J. 1993, ApJ, 412, 752, doi: 10.1086/172958

—. 1996, ApJ, 464, 943, doi: 10.1086/177381

Irwin, A. W. 2004, The FreeEOS Code for Calculating the Equation of State for Stellar Interiors. http://freeeos.sourceforge.net/

Itoh, N., Hayashi, H., Nishikawa, A., & Kohyama, Y. 1996, ApJS, 102, 411, doi: 10.1086/192264

Jermyn, A. S., Anders, E. H., Lecoanet, D., & Cantiello, M. 2022a, ApJS, 262, 19, doi: 10.3847/1538-4365/ac7cee

Jermyn, A. S., Schwab, J., Bauer, E., Timmes, F. X., & Potekhin, A. Y. 2021, ApJ, 913, 72, doi: 10.3847/1538-4357/abf48e

Jermyn, A. S., Bauer, E. B., Schwab, J., et al. 2022b, arXiv e-prints, arXiv:2208.03651, doi: 10.48550/arXiv.2208.03651

Landau, L. D., & Lifshitz, E. M. 1987, Fluid Mechanics

Langanke, K., & Martínez-Pinedo, G. 2000, Nuclear Physics A, 673, 481, doi: 10.1016/S0375-9474(00)00131-7

Le Saux, A., Baraffe, I., Guillet, T., et al. 2023, MNRAS, doi: 10.1093/mnras/stad1067

Le Saux, A., Guillet, T., Baraffe, I., et al. 2022, A&A, 660, A51, doi: 10.1051/0004-6361/202142569

Lecoanet, D., Cantiello, M., Anders, E. H., et al. 2021, MNRAS, 508, 132, doi: 10.1093/mnras/stab2524

Lecoanet, D., Le Bars, M., Burns, K. J., et al. 2015, PhRvE, 91, 063016, doi: 10.1103/PhysRevE.91.063016

Lecoanet, D., & Quataert, E. 2013, MNRAS, 430, 2363, doi: 10.1093/mnras/stt055

Lecoanet, D., Vasil, G. M., Burns, K. J., Brown, B. P., & Oishi, J. S. 2019, Journal of Computational Physics: X, 3, 100012, doi: https://doi.org/10.1016/j.jcpx.2019.100012

Lecoanet, D., Cantiello, M., Quataert, E., et al. 2019, ApJL, 886, L15, doi: 10.3847/2041-8213/ab5446

Mathis, S., Neiner, C., & Tran Minh, N. 2014, A&A, 565, A47, doi: 10.1051/0004-6361/201321830

Oda, T., Hino, M., Muto, K., Takahara, M., & Sato, K. 1994, Atomic Data and Nuclear Data Tables, 56, 231, doi: 10.1006/adnd.1994.1007

Oishi, J., Burns, K., Clark, S., et al. 2021, The Journal of Open Source Software, 6, 3079, doi: 10.21105/joss.03079

Paxton, B., Bildsten, L., Dotter, A., et al. 2011, ApJS, 192, 3, doi: 10.1088/0067-0049/192/1/3

Paxton, B., Cantiello, M., Arras, P., et al. 2013, ApJS, 208, 4, doi: 10.1088/0067-0049/208/1/4

Paxton, B., Marchant, P., Schwab, J., et al. 2015, ApJS, 220, 15, doi: 10.1088/0067-0049/220/1/15

Paxton, B., Schwab, J., Bauer, E. B., et al. 2018, ApJS, 234, 34, doi: 10.3847/1538-4365/aaa5a8

Paxton, B., Smolec, R., Schwab, J., et al. 2019, ApJS, 243, 10, doi: 10.3847/1538-4365/ab2241

Pope, S. B. 2000, Turbulent Flows

Potekhin, A. Y., & Chabrier, G. 2010, Contributions to Plasma Physics, 50, 82, doi: 10.1002/ctpp.201010017

Poutanen, J. 2017, ApJ, 835, 119, doi: 10.3847/1538-4357/835/2/119

Ratnasingam, R. P., Edelmann, P. V. F., & Rogers, T. M. 2019, MNRAS, 482, 5500, doi: 10.1093/mnras/sty3086

Ricker, G. R., Winn, J. N., Vanderspek, R., et al. 2014, Journal of Astronomical Telescopes, Instruments, and Systems, 1, 014003, doi: 10.1117/1.JATIS.1.1.014003

Rogers, F. J., & Nayfonov, A. 2002, ApJ, 576, 1064, doi: 10.1086/341894

Rogers, T. M., Lin, D. N. C., McElwaine, J. N., & Lau, H. H. B. 2013, ApJ, 772, 21, doi: 10.1088/0004-637X/772/1/21

Saumon, D., Chabrier, G., & van Horn, H. M. 1995, ApJS, 99, 713, doi: 10.1086/192204

Sullivan, C., & Kaszynski, A. 2019, The Journal of Open Source Software, 4, 1450, doi: 10.21105/joss.01450

Timmes, F. X., & Swesty, F. D. 2000, ApJS, 126, 501, doi: 10.1086/313304

Vasil, G. M., Julien, K., & Featherstone, N. A. 2021, Proceedings of the National Academy of Science, 118, e2022518118, doi: 10.1073/pnas.2022518118

Vasil, G. M., Lecoanet, D., Burns, K. J., Oishi, J. S., & Brown, B. P. 2019, Journal of Computational Physics: X, 3, 100013, doi: https://doi.org/10.1016/j.jcpx.2019.100013

Wang, D., & Ruuth, S. J. 2008, Journal of Computational Mathematics, 26, 838. http://www.jstor.org/stable/43693484

Weiss, A., Hillebrandt, W., Thomas, H. C., & Ritter, H. 2004, Cox and Giuli's Principles of Stellar Structure
